# Association between iron homestasis and all-cause mortality in acute pancreatitis: A retrospective MIMIC-IV database analysis

**DOI:** 10.1097/MD.0000000000046488

**Published:** 2025-12-12

**Authors:** Ting Yu, Feng Yi, Hongming Liu

**Affiliations:** a Department of Hepatobliliary Surgery, Daping Hospital, Army Medical University, Chongqing, China.

**Keywords:** acute pancreatitis, all-cause mortality, iron homeostasis, MIMIC-IV database, mortality

## Abstract

Iron is a vital trace element necessary for the human body, playing a key role in cell production, oxidative phosphorylation, and redox processes. However, research on iron homeostasis markers and prognosis in acute pancreatitis patients remains limited. This retrospective cohort study utilized data from the Medical Information Mart for Intensive Care-IV database. The primary outcome was 365-day all-cause mortality, while secondary outcomes included in-hospital, 30-day, and 90-day mortality. The relationships between iron homeostasis indicators (serum iron, ferritin, transferrin, and total iron binding capacity [TIBC]) and outcomes were analyzed using Kaplan–Meier survival curves, multivariable Cox proportional hazards models, and restricted cubic splines to explore nonlinear relationships. A total of 360 participants were included. The average age was 55.00 (44.00, 68.00) years, with 160 (44%) being females. In patients with acute pancreatitis admitted to the intensive care unit, Kaplan–Meier analysis showed that while higher serum iron and log_2_-ferritin were linked to increased all-cause mortality at all time points (in-hospital, 30, 90, and 365 days), lower transferrin and TIBC were associated with increased all-cause mortality only at the 30, 90, and 365-day follow-ups (log-rank *P* < .001). In the Cox regression model, continuous log_2_-ferritin was identified as a significant risk factor for 365-day all-cause mortality, with a hazard ratio of 1.157 per unit increase (95% confidence interval: 1.021–1.311, *P* = .022). In contrast, both as continuous variables and in quartile analysis, elevated transferrin and TIBC were significantly associated with a lower 365-day all-cause mortality risk. Furthermore, restricted cubic splines analysis revealed an S-shaped relationship for both transferrin and TIBC with in-hospital and 90-day all-cause mortality. In addition, subgroup analyses revealed a significant gender-dependent association for log_2_-ferritin with in-hospital and 90-day all-cause mortality, alongside interactions of transferrin with both ethnicity and body mass index, and of TIBC with ethnicity for all-cause in-hospital mortality. This study highlighted a significant link between iron homeostasis indicators and all-cause mortality risk in intensive care unit patients with AP. Larger prospective studies are required for further validation.

## 1. Introduction

Acute pancreatitis (AP) is an inflammatory condition caused by the premature activation of trypsinogen in pancreatic acinar cells and is a leading gastrointestinal reason for hospitalization worldwide.^[1]^ Over the past 2 decades, the global incidence of AP has risen steadily, resulting in substantial healthcare expenditures and socioeconomic burdens.^[2]^ Approximately 20% of patients progress to moderately severe or severe AP, characterized by devastating complications including pancreatic/peripancreatic necrosis and multiorgan dysfunction syndrome, which collectively contribute to poor prognoses with mortality rates exceeding 30% in severe cases.^[3–5]^ Given this clinical features, identification of robust prognostic determinants is critical for risk stratification and personalized therapeutic interventions.

Iron is an essential mineral that acts as a vital nutrient and regulator in cellular physiology, participating in oxygen transport, energy metabolism, cell proliferation/differentiation, and immune defense.^[6]^ The human body maintains iron homeostasis through sophisticated regulatory mechanisms. Dysregulation of iron metabolism, either deficiency or overload, can lead to systemic damage.^[7]^ Iron deficiency reduces hemoglobin synthesis and impairs immune function.^[8,9]^ Conversely, iron overload saturates transferrin, leading to non-transferrin-bound iron (NTBI) that generates reactive oxygen species through Fenton reactions, causing lipid peroxidation, cellular damage, immune dysfunction, and increased susceptibility to oxidative stress-related diseases and infections.^[10–12]^

In AP, impaired digestive function may disrupt iron homeostasis.^[13,14]^ Preclinical studies suggest that iron overload exacerbates pancreatic injury through oxidative damage in murine models.^[15]^ Serum iron is a widely used clinical marker for assessing iron homeostasis.^[16]^ Existing evidence supports serum iron as a prognositc marker for mortality in AP.^[17]^ Additional iron homeostasis indicators, including total iron binding capacity (TIBC), ferritin, and transferrin, have been explored for their clinical utility.^[18–20]^ However, the prognostic significance of TIBC, transferrin, and ferritin in AP remains unclear, with limited systematic investigations exploring iron homeostasis biomarkers and AP outcomes.

This study analyzed iron homeostasis indicators (serum iron, ferritin, TIBC, transferrin) and clinical data from AP patients in the Medical Information Mart for Intensive Care-IV (MIMIC-IV) database to evaluate their associations with in-hospital, 30-day, 90-day, and 365-day mortality. Our findings aim to clearify the prognostic value of iron homeostasis markers in AP management.

## 2. Material and methods

### 2.1. Database

This retrospective study analyzed data from individuals with AP in the MIMIC-IV database, a comprehensive resource developed and maintained by the Laboratory for Computational Physiology at the Massachusetts Institute of Technology. The database includes a large volume of high-quality patient medical records from the intensive care unit of Beth Israel Deaconess Medical Center from 2008 to 2019.^[21]^ Consent was waived since the database contained no protected health information and patients were anonymized.

### 2.2. Ethics statement

The MIMIC database was approved by the institutional review boards of Beth Israel Deaconess Medical Center (2001-P001699/14) and the Massachusetts Institute of Technology (No. 0403000206), waiving individual patient consent and permitting data sharing. Since the MIMIC-IV database is publicly accessible, this study did not require informed consent or additional ethical approval.

### 2.3. Study population and definitions

Intensive care unit (ICU) hospitalization records of patients diagnosed with AP from the MIMIC database were retrospectively selected based on International Classification of Disease, 9^th^ Revision code 577.0 or 10^th^ Revision code K85. Only data from the first ICU admission were analyzed for patients with multiple admissions. The primary inclusion criterion was the availability of measurements for serum iron, ferritin, transferrin, and TIBC within the first 24 hours of ICU admission. Patients under 18 at first admission and those without iron homeostasis data were excluded. In total, 752 patients were excluded due to the absence of complete iron homeostasis data. Consequently, a total of 360 patients were included in the study.

### 2.4. Data extraction

PostgresSQL (V14.6) and Navicate Premium (V16) tools were leveraged to extract data by running the Structured Query Language command. The extracted potential variabels consisted of: demographics, such as age, gender, ethnicity, and body mass index (BMI); laboratory results, including ferritin, serum iron, transferrin level, TIBC, alanine aminotransferase (ALT) levels, glucose, white blood cell count, platelet count, creatinine, and anion gap; and comorbidities at baseline, including hypertension, sepsis, heart failure, diabetes, chronic pulmory disease, simplified acute physiology score II (SAPS II), malignant cancer, Charlson comorbidity index (CCI), and sequential organ failure assessment (SOFA) score.

Follow-up started on the day of admission and ended on the day of death. In this study, due to substantial positive skewness in the distribution of ferritin levels, ferritin was log-transformed for analysis. All laboratory variables and disease severity scoring were derived from the first admission data.

To minimize bias, covariates with more than 25% missing data were excluded from the analysis. Missing data for the remaining covariates were handled using multiple imputation by chained equations with the “mice” package in R.

### 2.5. Outcomes

The outcome of patients with AP in MIMIC-IV database includes in-hospital all-cause mortality, 30-day all-cause mortality, 90-day all-cause mortality, 365-day all-cause mortality. The primary outcomes of present study were 365-day all-cause mortality. Secondary outcomes were in-hospital all-cause mortality, 30-day all-cause mortality, and 90-day all-cause mortality.

### 2.6. Statistical method

Continuous variables were reported as mean ± standard deviation or median with IQR based on distribution, while categorical variables were presented as percentages. The Kolmogorov–Smirnov test was leveraged to discern the normality of continuous data. The *t*-test or analysis of variance was used for normally distributed continuous variables, while the Mann–Whitney or Kruskal–Wallis test was applied to non-normally distributed data. Kaplan–Meier analysis evaluated outcome incidence across iron homeostasis indicators (serum iron, log_2_-ferritin, transferrin, and TIBC), with differences tested by the log-rank test. The Cox model estimated hazard ratios (HRs) and 95% confidence interval (CI) for associations between iron homeostasis indicators and outcomes. The model was adjusted in steps for confounders: Model 1 (unadjusted), Model 2 (adjusted for age, gender, ethnicity, and BMI), and Model 3 (adjusted for age, gender, ethnicity, BMI, ALT, creatinine, white blood cell, anion gap, platelets, glucose, hypertension, sepsis, heart failure, diabetes, chronic pulmonary disease, SAPS II, malignant cancer, CCI, and SOFA). Restricted cubic splines (RCS) were applied to assess nonlinear relationships between iron homeostasis indicators and mortality risk. All statistical analyses were performed using R software (V4.1), with a *P*-value < .05 regarded significant.

## 3. Results

### 3.1. Baseline characteristics of study individual

**Figure 1.**
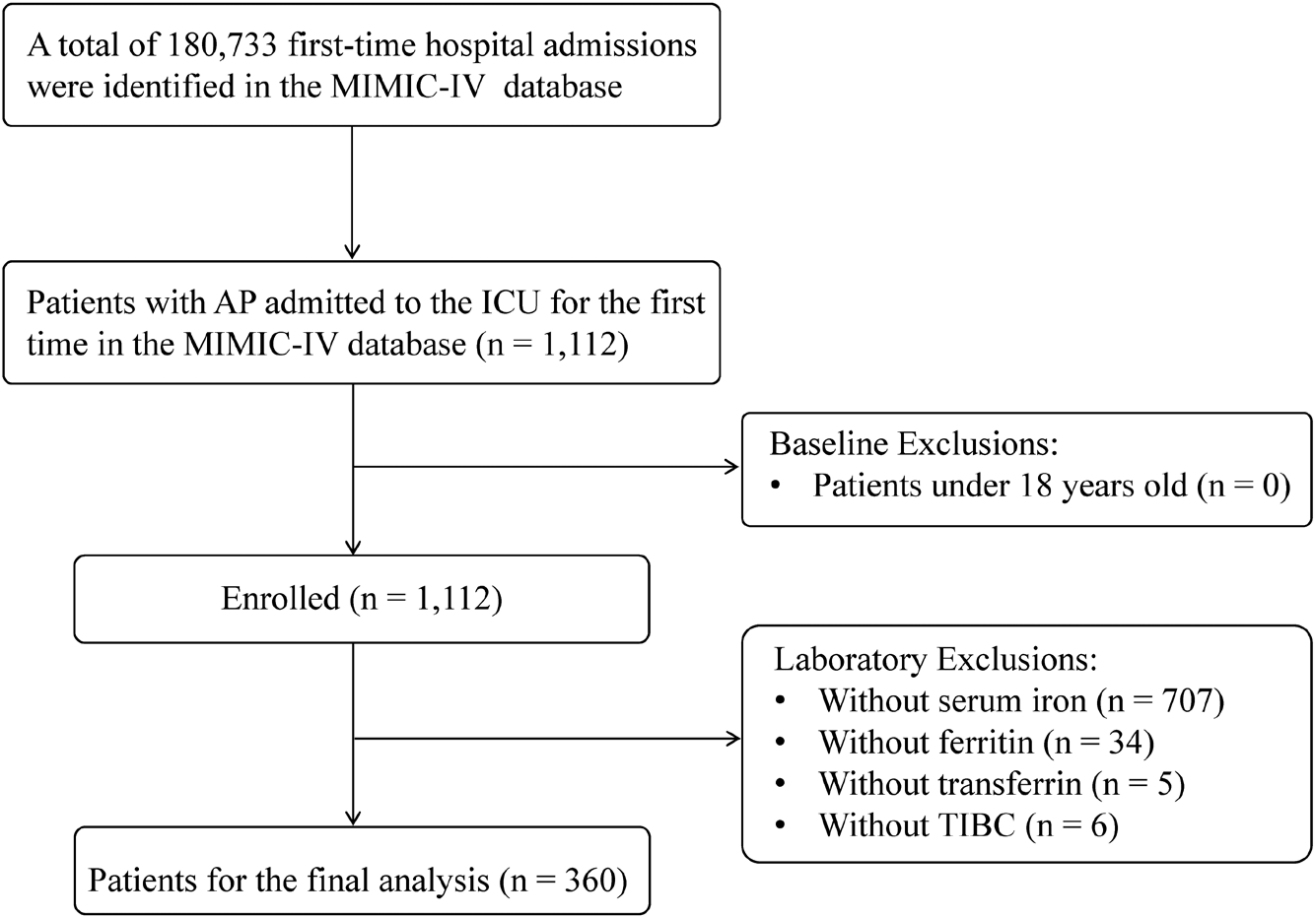
Study cohort flowchart.

This study analyzed 360 AP patients, with the flowchart presented in Figure 1.

The median patient age was 55 years (IQR, 44–68), with 56% male and over 60% White. Baseline characteristics are shown in Table 1. Compared with survivors, non-survivors had significantly higher creatinine levels, heart failure prevalence, SOFA, CCI, and SAPS II scores, as well as a higher incidence of malignant cancer, while transferrin, TIBC, and hypertension rates were significantly lower. No significant differences were found in BMI, length of stay, serum iron, log_2_-transformed ferritin, platelet count, glucose, ALT, anion gap, diabetes, chronic pulmonary disease, or sepsis. Mortality rates were 15% in-hospital, 11% at 30 days, 21% at 90 days, and 27% at 365 days.

**Table 1:** Baseline characteristics of the study cohort.

Characteristic	Overall, N = 360	Survivors, N = 231	Non-survivors, N = 129	*P*-value*
Demographics				
Age (yr), Median (IQR)	55.00 (44.00, 68.00)	52.00 (40.50, 63.50)	64.00 (50.00, 75.00)	<.001
Gender, n (%)				.090
Female	160 (44%)	95 (41%)	65 (50%)	
Male	200 (56%)	136 (59%)	64 (50%)	
Ethnicity, n (%)				.70
Asian	12 (3.3%)	7 (3.0%)	5 (3.9%)	
Black	39 (11%)	24 (10%)	15 (12%)	
Others	87 (24%)	60 (26%)	27 (21%)	
White	222 (62%)	140 (61%)	82 (64%)	
BMI (kg/m^2^), Median (IQR)	28.65 (24.37, 33.78)	28.69 (24.23, 34.54)	28.57 (24.40, 31.81)	.20
Length of initial stay, Median (IQR)	20.69 (11.81, 36.83)	20.26 (11.49, 32.38)	21.71 (13.20, 41.78)	.20
Laboratory data				
Ferritin (log_2_), ng/mL, Median (IQR)	9.46 (8.42, 10.49)	9.50 (8.59, 10.42)	9.34 (8.04, 10.75)	.80
Transferrin, mg/dL, Median (IQR)	136.00 (98.00, 168.25)	139.00 (104.00, 171.50)	131.00 (93.00, 159.00)	.032
Serum iron, μg/dL, Median (IQR)	35.00 (20.00, 62.00)	35.00 (20.00, 59.00)	37.00 (21.00, 67.00)	.20
TIBC, μg/dL, Median (IQR)	177.00 (127.00, 218.50)	181.00 (135.00, 223.00)	170.00 (121.00, 207.00)	.03
WBC, K/μL, Median (IQR)	12.80 (8.88, 19.13)	12.80 (9.05, 19.05)	12.90 (7.90, 19.20)	.70
Platelet, K/μL, Median (IQR)	193.00 (129.25, 280.25)	195.00 (134.00, 281.50)	190.00 (122.00, 274.00)	.50
Glucose, mg/dL, Median (IQR)	134.50 (104.75, 179.00)	135.00 (101.00, 186.00)	132.00 (110.00, 177.00)	.90
ALT, IU/L, Median (IQR)	53.00 (23.75, 126.25)	55.00 (23.00, 146.00)	49.00 (24.00, 97.00)	.40
Creatinine, mg/dL, Median (IQR)	1.30 (0.78, 2.70)	1.10 (0.70, 2.20)	1.60 (0.80, 3.40)	.003
Anion gap, Median (IQR)	16.00 (13.00, 20.00)	16.00 (12.00, 19.00)	17.00 (13.00, 21.00)	.063
Comorbidities				
Heart failure, n (%)	62 (17%)	30 (13%)	32 (25%)	.004
Hypertension, n (%)	115 (32%)	88 (38%)	27 (21%)	<.001
Diabetes, n (%)	42 (12%)	29 (13%)	13 (10%)	.50
SOFA, Median (IQR)	7.00 (3.00, 10.00)	6.00 (3.00, 9.00)	8.00 (4.00, 11.00)	.002
CCI, Median (IQR)	4.00 (2.00, 6.00)	3.00 (1.00, 5.00)	5.00 (3.00, 7.00)	<.001
SAPS II, Median (IQR)	39.00 (28.00, 50.25)	34.00 (25.50, 46.50)	44.00 (35.00, 56.00)	<.001
Chronic pulmonary disease, n (%)	73 (20%)	48 (21%)	25 (19%)	.80
Malignant cancer, n (%)	30 (8.3%)	11 (4.8%)	19 (15%)	.001
Sepsis, n (%)	268 (74%)	168 (73%)	100 (78%)	.30
Outcome				
Hospital mortality, n (%)	55 (15%)	-	55 (43%)	<.001
7-d mortality, n (%)	6 (1.7%)	-	6 (4.7%)	.002
30-d mortality, n (%)	41 (11%)	-	41 (32%)	<.001
90-d mortality, n (%)	76 (21%)	-	76 (59%)	<.001
365-d mortality, n (%)	97 (27%)	-	97 (75%)	<.001

Continuous variables are presented as mean ± SD if normally distributed, and median (interquartile range) if not normally distributed. Categorical variables are presented as number of patients (%).

ALT = alanine aminotransferase, BMI = body mass index, CCI = Charlson comorbidity index, SAPS II = simplified acute physiology score II, SD = standard deviation, SOFA = sequential organ failure assessment, TIBC = total iron binding capacity, WBC = white blood cell.

* Wilcoxon rank sum test; Pearson Chi-squared test; Fisher exact test.

To investigate the prognostic significance of iron metabolism markers (serum iron, log_2_-transformed ferritin, transferrin, and TIBC), we compared their baseline levels between survivor and non-survivor groups at in-hospital, 30-day, 90-day, and 365-day follow-ups. Survivors consistently demonstrated that significantly lower baseline serum iron levels compared non-survivors at the in-hospital, 30-day, and 90-day assessments (Fig. S1A, Supplemental Digital Content, https://links.lww.com/MD/Q898). Similarly, log_2_-ferritin levels were significantly lower in patients who survived at the in-hospital and 30-day time points (Fig. S1B, Supplemental Digital Content, https://links.lww.com/MD/Q898). In contrast, transferrin and TIBC were significantly higher in survivors across all assessed time points (Fig. S1C and D, Supplemental Digital Content, https://links.lww.com/MD/Q898).

### 3.2. Kaplan–Meier analysis

**Figure 2.**
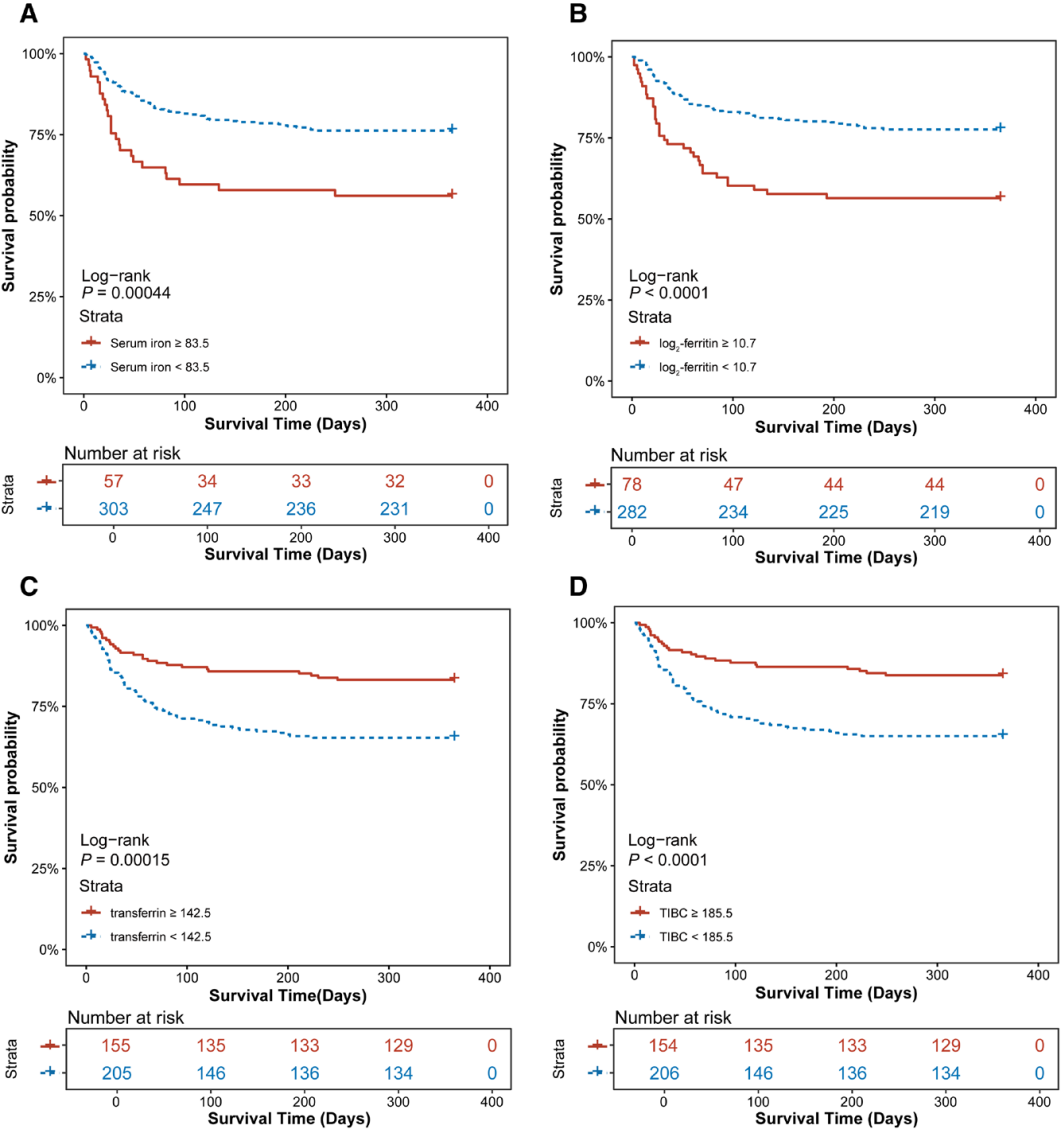
KM survival curves depicting the association between iron homeostasis indicators and 365-day mortality (A, serum iron; B, log_2_-Ferritin; C, transferrin; D, TIBC). TIBC = total iron binding capacity.

Patients were classified into high- and low-risk groups using optimal cutoff values for iron homeostasis indicators, determined by receiver operating characteristic curve analysis. The Kaplan–Meier survival analysis was used to assess the incidence of primary or secondary endpoints across groups. As shown in Figure 2, higher serum iron (log-rank *P *< .001), higher log_2_-ferritin (log-rank *P *< .001), lower tansferrin (log-rank *P *< .001), and lower TIBC (log-rank *P *< .001) were significantly linked to increased 365-day mortality in AP patients. Patients with higher serum iron and log_2_-ferritin had increased in-hospital mortality. Similarly, higher serum iron, higher log_2_-ferritin, lower transferrin, and lower TIBC were associated with increased 30- and 90-day mortality. However, no significant differences were observed in in-hospital Kaplan–Meier survival curves between AP patients stratified by transferrin (log-rank *P = *.058) and TIBC (log-rank *P = *.1). Figures S2 to S4, Supplemental Digital Content, https://links.lww.com/MD/Q898, show the Kaplan–Meier survial analysis results for in-hospital, 30-day, and 90-day mortality.

### 3.3. Association of Iron Homeostasis with Outcomes in Acute AP

Cox proportional hazards models in Model 3 evaluated the relationships between iron homeostasis indicators (serum iron, log_2_-ferritin, transferrin, and TIBC) and mortality endpoints (in-hospital, 30-day, 90-day, and 365-day) in AP patients.

Elevated serum iron levels emerged as a consistent risk factor across multiple mortality endpoints. When analyzed as a continuous variables, each unit increase in serum iron was assocated with a 0.6% higher risk of in-hospital mortality (HR = 1.006, 95% CI: 1.000–1.012; *P* = .040; Table S1, Supplemental Digital Content, https://links.lww.com/MD/Q898), 0.8% for 30-day mortality (HR = 1.008, 95% CI: 1.002–1.014; *P* = .012; Table S1, Supplemental Digital Content, https://links.lww.com/MD/Q898), and 0.6% for 90-day mortality (HR = 1.006, 95% CI: 1.001–1.010; *P* = .021; Table S1, Supplemental Digital Content, https://links.lww.com/MD/Q898). Categorical analysis further revealed that patients in the fourth quartile (Q4) levels of serum iron faced over fourth the risk of 30-day mortality risk (HR = 4.098, 95% CI: 1.247–13.464; *P *= .020; Table S1, Supplemental Digital Content, https://links.lww.com/MD/Q898), and 2.6-fold higher 90-day mortality risk (HR = 2.603, 95% CI: 1.222–5.542; *P* = .013; Table S1, Supplemental Digital Content, https://links.lww.com/MD/Q898) compared with the first quartile (Q1). Similarly, log_2_-ferritin levels demonstrated significant prognostic value. Continuous analysis identified a 28.1% increased 30-day mortality risk (HR = 1.281, 95% CI: 1.051–1.561; *P *= .014; Table S2, Supplemental Digital Content, https://links.lww.com/MD/Q898) and 16.8% higher 365-day mortality risk (HR: 1.157, 95% CI: 1.021–1.311; *P *= .022; Table 2) per log_2_-unit increment.

**Table 2:** Cox proportional hazard models for iron homeostasis indicators and 365-day mortality in AP patients.

Iron homeostasis indicators	Model 3	
	HR (95% CI)	*P*-value
Serum iron		
Continuous variable per unit (μg/dL)	1.004 (0.999–1.008)	.101
Quartile		
Q1	Ref.	
Q2	1.075 (0.576–2.008)	.820
Q3	0.815 (0.418–1.589)	.547
Q4	1.725 (0.915–3.253)	.092
log_2_-Ferritin		
Continuous variable per unit, original unit (ng/mL)	1.157 (1.021–1.311)	**.022**
Quartile		
Q1	Ref.	
Q2	1.156 (0.627–2.13)	.643
Q3	0.802 (0.416–1.548)	.511
Q4	1.701 (0.956–3.025)	.071
Transferrin		
Continuous variable per unit (mg/dL)	0.994 (0.989–0.998)	**.007**
Quartile		
Q1	Ref.	
Q2	0.837 (0.491–1.426)	.513
Q3	0.666 (0.370–1.199)	.175
Q4	0.343 (0.179–0.658)	**.001**
Total iron binding capacity		
Continuous variable per unit (μg/dL)	0.995 (0.992–0.999)	**.007**
Quartile		
Q1	Ref.	
Q2	0.858 (0.506–1.454)	.569
Q3	0.640 (0.352–1.161)	.142
Q4	0.343 (0.179–0.657)	**.001**

Model 3: adjusted for gender, age, race, BMI, WBC, platelet, glucose, ALT, creatinine, anion gap, heart failure, hypertension, diabetes, SOFA, CCI, SAPS II, chronic pulmonary disease, malignant cancer, and spesis. Bold values indicate that the *P*-value is statistically significant.

ALT = alanine aminotransferase, BMI = body mass index, CCI = Charlson comorbidity index, CI = confidence interval, HR = hazard ratio, SAPS II = simplified acute physiology score II, SOFA = sequential organ failure assessment, TIBC = total iron binding capacity, WBC = white blood cell.

In contrast, transferrin and TIBC exhibited protective effects. Continuous transferrin levels were inversely associated with 30-day (HR = 0.992, 95% CI: 0.985–1.000; *P *= .041; Table S3, Supplemental Digital Content, https://links.lww.com/MD/Q898), 90-day (HR = 0.995, 95% CI: 0.990–1.000; *P *= .039; Table S3, Supplemental Digital Content, https://links.lww.com/MD/Q898), and 365-day mortality (HR = 0.994, 95% CI: 0.989–0.998; *P *= .007; Table 2). Patients in the highest transferrin quartile (Q4) had significantly reduced mortality risks compared with Q1: 81.4% lower for 30-day (HR = 0.186, 95% CI: 0.053–0.653; *P *= .009; Table S3, Supplemental Digital Content, https://links.lww.com/MD/Q898), 69.6% for 90-day (HR = 0.304, 95% CI: 0.138–0.672; *P *= .003; Table S3), and 65.7% for 365-day mortality (HR = 0.343, 95% CI: 0.179–0.658; *P *= .002;, Supplemental Digital Content, https://links.lww.com/MD/Q898, Table 2). TIBC also exhibited a protective pattern, being inversely associated with 30-day (HR = 0.994, 95% CI: 0.988–1; *P *= .041; Table S4, Supplemental Digital Content, https://links.lww.com/MD/Q898), 90-day (HR = 0.996, 95% CI: 0.992–1; *P *= .038; Table S4, Supplemental Digital Content, https://links.lww.com/MD/Q898), and 365-day mortality (HR = 0.995; 95% CI: 0.992–0.999; *P* = .007; Table 2). Furthermore, the Q4 levels of TIBC were associated with 81.3% lower 30-day mortality (HR = 0.187, 95% CI: 0.053–0.655; *P *= .009; Table S4, Supplemental Digital Content, https://links.lww.com/MD/Q898), 69.6% lower 90-day mortality (HR = 0.304, 95% CI: 0.138–0.672; *P *= .003; Table S4, Supplemental Digital Content, https://links.lww.com/MD/Q898), and 65.7% lower 365-day mortality (HR = 0.343, 95% CI: 0.179–0.657; *P *= .001; Table 2) relative to Q1. Additionally, to ensure the robustness of our results, we performed sensitivity analyses using both a stepwise logistic regression and alternative imputation methods. Neither of these approaches altered our main findings, confirming the stability of our findings (Tables S5–S12, Supplemental Digital Content, https://links.lww.com/MD/Q898).

### 3.4. The detection of nonlinear relationships

**Figure 3.**
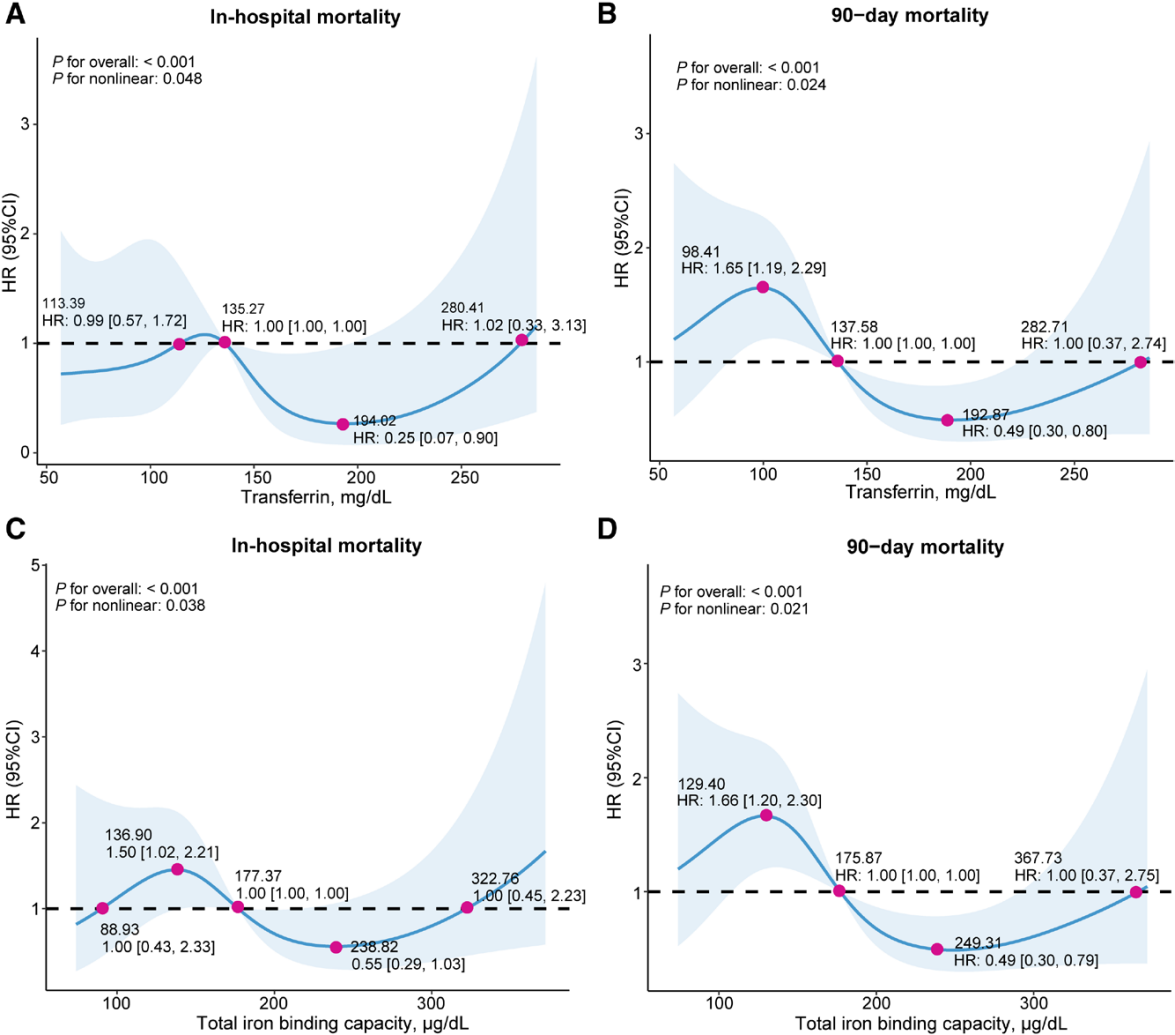
Restricted cubic spline curves illustrate the adjusted hazard ratios for the association of serum transferrin with (A) in-hospital and (B) 90-day mortality, and total iron binding capacity (TIBC) with (C) in-hospital and (D) 90-day mortality. The blue lines represent the estimated adjusted hazard ratios, while the blue band indicates the corresponding 95% confidence interval. Adjustments follow Model 3.

RCS were used to illustrate the nonlinear relationship between iron homeostasis indicators and in-hospital outcomes, 30-day, 90-day, and 365-day all-cause mortality (Figs. 3 and S5–S8, Supplemental Digital Content, https://links.lww.com/MD/Q898). Both transferrin and TIBC exhibited approximately S-shaped associations with in-hospital and 90-day all-cause mortality (Figs. 3A–D). For transferrin, both low and high levels of transferrin were associated with an increased risk of mortality. For 90-day mortality (Fig. 3B), the risk was lowest at transferrin levels of 192.87 mg/dL (HR = 0.49, 95% CI: 0.30–0.80). As transferrin levels decreased, the risk increased significantly. Notably, at 98.41 mg/dL, the HR was 1.65 (95% CI: 1.19–2.29). The risk also trended upward at very high transferrin levels, albeit with wide confidence intervals. A similar S-shaped pattern was observed for in-hospital mortality (Fig. 3A).

Likewise, TIBC demonstrated a biphasic pattern. For 90-day mortality, the risk reached its nadir at a TIBC level of ~249 µg/dL (HR: 0.49, 95% CI: 0.30–0.79), suggesting a protective effect at this intermediate range. Conversely, lower TIBC were strong associated with increased mortality, with an HR of 1.66 (95% CI: 1.20–2.30) at a TIBC of 129.4 µg/dL (Fig. 3D). This S-shaped relationship was also consistent for in-hospital mortality (Fig. 3C).

### 3.5. Subgroups analysis

To investigate whether the associations between iron homeostasis indicators and mortality endpoints (in-hospital, 30-day, 90-day, and 365-day) varied across different conditions, subgroup analyses were performed for gender, age, ethnicity, and BMI.

The association between iron homeostasis indicators and 30- and 365-day mortality remained consistent across all strata (*P* for interaction > .05, Figs. S10 and S12, Supplemental Digital Content, https://links.lww.com/MD/Q898). However, for in-hospital mortality, interactions were observed between log_2_-ferritin and gender, of transferrin with both ethnicity and BMI, and between TIBC and ethnicity. The correlation between log_2_-ferritin and in-hospital all-cause mortality was statistically significant only in the male subgroup. Additionally, the association between transferrin and in-hospital mortality was significant only in the BMI ≥ 30 and others ethnicity subgroups, whereas for TIBC, this association was significant only in the others ethnicity subgroup (Fig. S9, Supplemental Digital Content, https://links.lww.com/MD/Q898).

For 90-day mortality, an interaction between log_2_-ferritin and gender was detected (Fig. S11, Supplemental Digital Content, https://links.lww.com/MD/Q898). Overall, the results indicate that the association between log_2_-ferritin and in-hospital and 90-day mortality is gender-dependent (*P* for interaction < .05), with a positive correlation being present only in males.

## 4. Discussion

This study is the first to independently link iron homeostasis indicators to clinical outcomes in ICU patients with AP. In the primary outcomes, elevated log_2_-ferritin levels were significantly associated with and increased risk of 365-day all-cause mortality in AP patients, while higher transferrin and TIBC levels were linked to reduced 365-day all-cause mortality risk. For secondary outcomes, elevated serum iron levels correlated with higher risks of in-hospital, 30-day, and 90-day all-cause mortality. Log_2_-ferritin was linked to a higher risk of 30-day mortality, while transferrin and TIBC were consistently associated with lower 30- and 90-day mortality. Furthermore, a near-S-shaped nonlinear relationship was observed between transferrin and TIBC levels and both in-hospital and 90-day mortality.

Previous study have indicated that serum iron is associated with an increased risk of mortality in patients with AP, with the average serum iron being significantly higher in non-survivor groups compared with survivors.^[17]^ Our study further substantiates that serum iron is independently associated with higher in-hospital, 30-day, and 90-day all-cause mortality. This association may be mechanistically explained by massive parenchymal cell destruction in moderate-to-severe AP, facilitating intracellular iron release into circulation.^[22,23]^ Serum ferritin is not only a storage protein that reflects body iron reserves but also an acute-phase reactant.^[24,25]^ Existing evidence supports its role in distinguishing mild from severe AP.^[26]^ Our study found that elevated ferritin levels were significantly associated with 30- and 365-day all-cause mortality in ICU patients with AP. The mechanisms behind ferritin elevation during inflammation in AP likely involve 2 aspects. On one hand, severe systemic inflammation triggers pro-inflammatory cytokines such as IL-1β and IL-6, which stimulate ferritin synthesis, causing a sharp increase in serum ferrtin.^[27,28]^ On the other hand, local inflammation and pancreatic tissue damage lead to necrosis of acinar cells, releasing large amounts of intracellular ferritin into circulation.^[29–31]^ Therefore, the high ferritin levels observed in ICU patients with AP are not just a marker of iron storage but likely reflect the combined effects of systemic inflammation and local tissue injury.

In contrast, transferrin and TIBC exhibited protective effects against 30-, 90-, and 365-day mortality. Transferrin, the primary iron transport protein, and TIBC collectvely regulate iron distribution.^[32]^ During inflammation, suppressed transferrin synthesis reduces TIBC, leading to NTBI accumulation and oxidative damage.^[33–35]^ Notably, early enteral nutrition may improve outcomes by increasing transferrin levels, suggesting a promising therapeutic strategy that warrants clinical validation.^[36,37]^

Furthermore, RCS analyses revealed dose–response relationships between transferrin and TIBC and both in-hospital and 90-day all-cause mortality in ICU patients with AP. Transferrin levels below the threshold of 137.58 mg/dL, linked to increased mortality risk, may be due to iron-restricted erythropoiesis and immune suppression.^[38,39]^ Conversely, levels above this threshold likely mitigate oxidative stress by sequestering catalytic iron.^[40]^ Similarly, lower TIBC reflects impaired iron buffering capacity, permitting NTBI accumulation and ferroptosis.^[41]^ In contrast, higher TIBC helps to maintain iron homeostasis and exerts protective effects.^[42,43]^ Subgroup analyses revealed interactions between iron homeostasis indicators and mortality risk. The gender-dependent association between log_2_-ferritin and in-hospital mortality as well as 90-day mortality, significant in males, may reflect gender-related differences in immune responses and inflammation in AP.^[44,45]^ Elevated transferrin reduce in-hospital mortality risk in high BMI patients, highlighting its dual role as an acute-phase reactant and obesity-related oxidative mediator.^[46,47]^ The observed ethnic differences in the protective effects of transferrin and TIBC should be interpreted cautiously, as they are likely confounded by baseline imbalances between the groups. In contrast, iron homeostasis indicators showed no interaction effects across subgroups for 30- and 365-day mortality, reinforcing their reliability as prognostic markers. Future studies should validate these thresholds in diverse populations and explore iron-modulating therapies for high-risk subgroups.

However, our study has several limitations. First, as a single-center study, it cannot establish causal relationships. Second, the selection of AP patients was based on International Classification of Disease codes in the MIMIC database, although coding inaccuracies and selection bias cannot be excluded. Third, the absence of long-term follow-up data in the MIMIC-IV database restricted our study to in-hospital, 30-day, 90-day, and 365-day mortality, potentially limiting the overall prognosis assessment. Fourth, despite extensive adjustment, residual confounding could still influence some of the observed associations. Fifth, we used multiple imputations to handle missing data and confirmed through sensitivity analyses with 3 imputation algorithms that the direction of the effect remained consistent. Nonetheless, potential variability from imputation warrants cautious interpretation of our findings. Furthermore, our findings are primarily applicable to ICU patients with severe AP and should be cautiously extrapolated to non-ICU cases. Further studies in broader AP populations are needed for validation.

## 5. Strengths and limitations of this study

This study employed multiple statistical mothods to investigate the relationship between iron homeostasis and all-cause mortality in acute pancreatitis, adjusting for various clinical variables to enhance the rigor of the analysis.

However, a limitation of this study is its retrospective design, which cannot establish a cause-and-effect relationship.

Moreover, the relatively small sample size means that larger studies are needed to validate the findings of this research.

## 6. Conclusion

This study established significant association between iron homeostasis indicators and clinical outcomes in AP patients, highlighting their potential as novel prognostic tools for short- and long-term mortality risk stratification. Regular monitoring of these markers could guide clinical decision-making, especially in identifying high-risk patients needing intensive care or targeted interventions. However, further research is needed on interventions targeting iron homeostasis to enhance the prognosis of AP patients.

## Acknowledgments

We sincerely thank the MIMIC-IV team and participants. Our appreciation also extends to the editors and reviewers for their rigorous feedback.

## Author contributions

**Conceptualization:** Feng Yi, Hongming Liu.

**Data curation:** Feng Yi.

**Funding acquisition:** Feng Yi, Hongming Liu.

**Investigation:** Ting Yu, Feng Yi.

**Methodology:** Ting Yu.

**Software:** Ting Yu.

**Supervision:** Feng Yi.

**Visualization:** Ting Yu.

**Writing – original draft:** Ting Yu.

**Writing – review & editing:** Ting Yu, Hongming Liu.

## Supplementary Material
