# Supplementary material for: Association between iron homestasis and all-cause mortality in acute pancreatitis: A retrospective MIMIC-IV database analysis
Source: Medicine (Baltimore). 2025 Dec 12;104(50):e46488. doi: 10.1097/MD.0000000000046488 (PMC12708177; doi:10.1097/MD.0000000000046488)
Supplement: Supplementary file 1 [file medi-104-e46488-s001.pdf]

Figure S1. Comparison of iron metabolism markers between survival and non-survival groups at different time points (**A**, Serum iron; **B**, log_2-Ferritin; **C**, Transferrin; **D**, TIBC). Abbreviations: TIBC, total iron binding capacity.

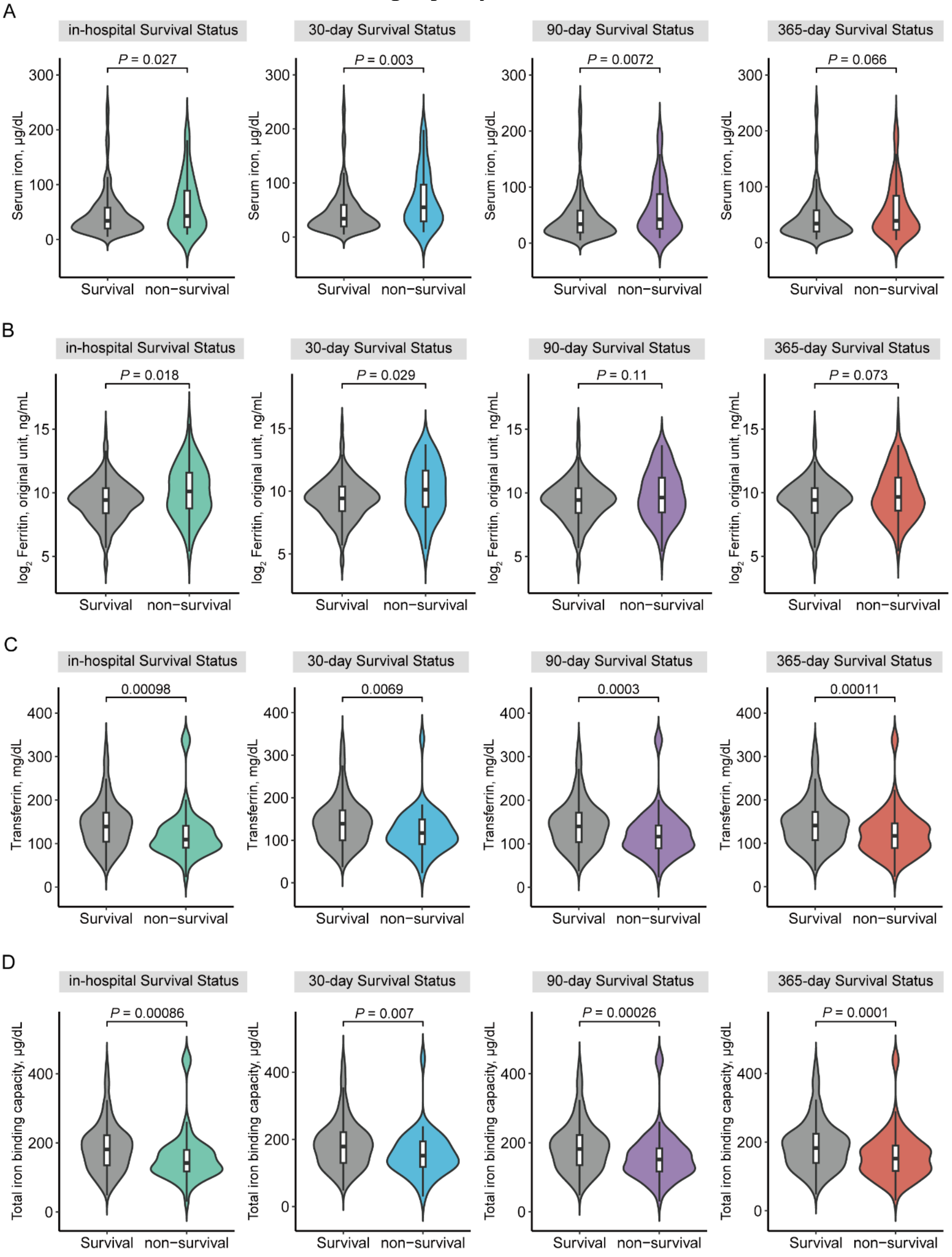

Figure S2. Kaplan-Meier survival analysis curves showing the relationship between iron homeostasis indicators and in-hospital all-cause mortality (**A**, Serum iron; **B**, log_2-Ferritin; **C**, Transferrin; **D**, TIBC). Abbreviations: TIBC, total iron binding capacity.

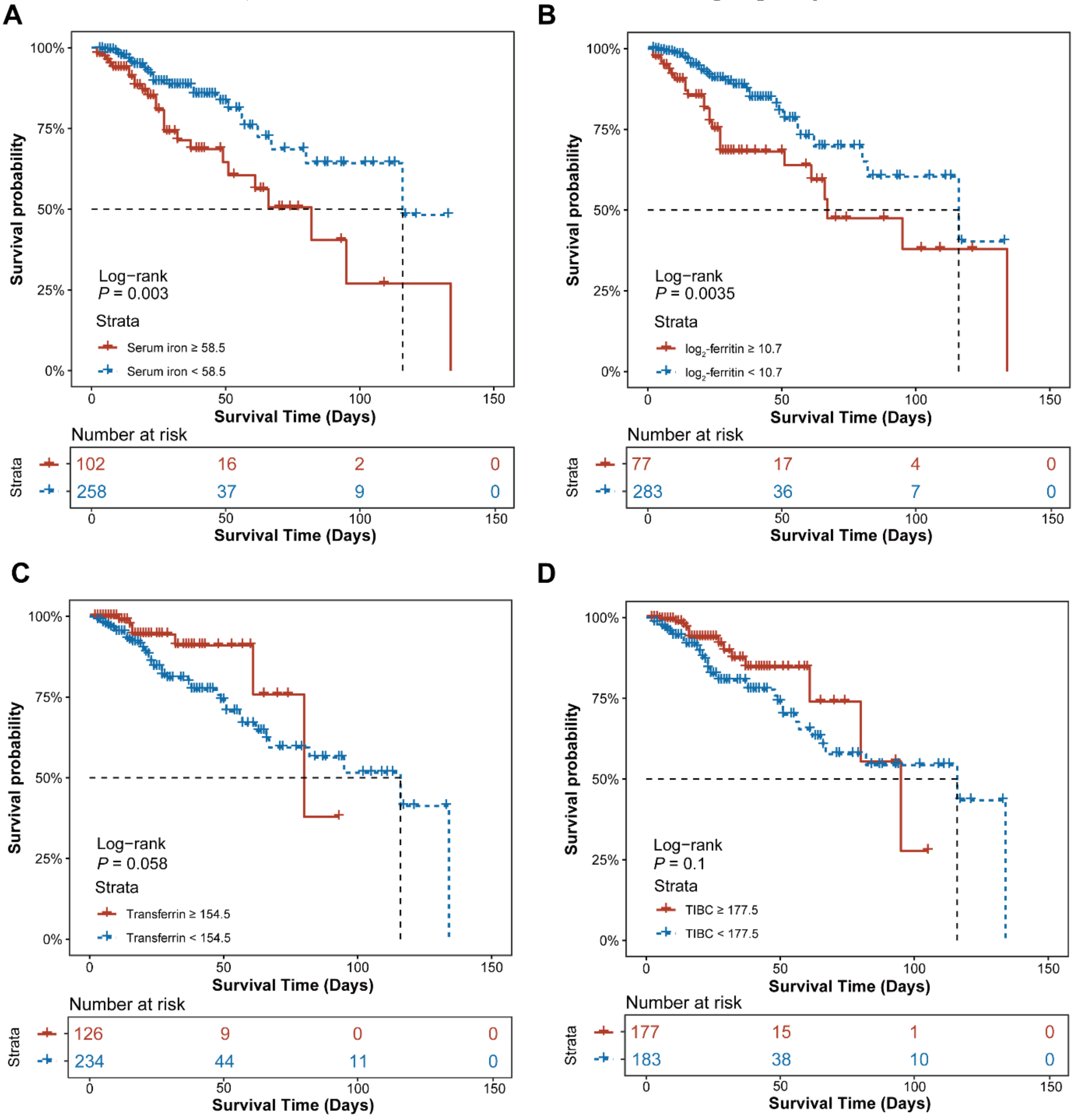

Figure S3. Kaplan-Meier survival analysis curves showing the relationship between iron homeostasis indicators and 30-day all-cause mortality (**A**, Serum iron; **B**, log2-Ferritin; **C**, Transferrin; **D**, TIBC). Abbreviations: TIBC, total iron binding capacity.

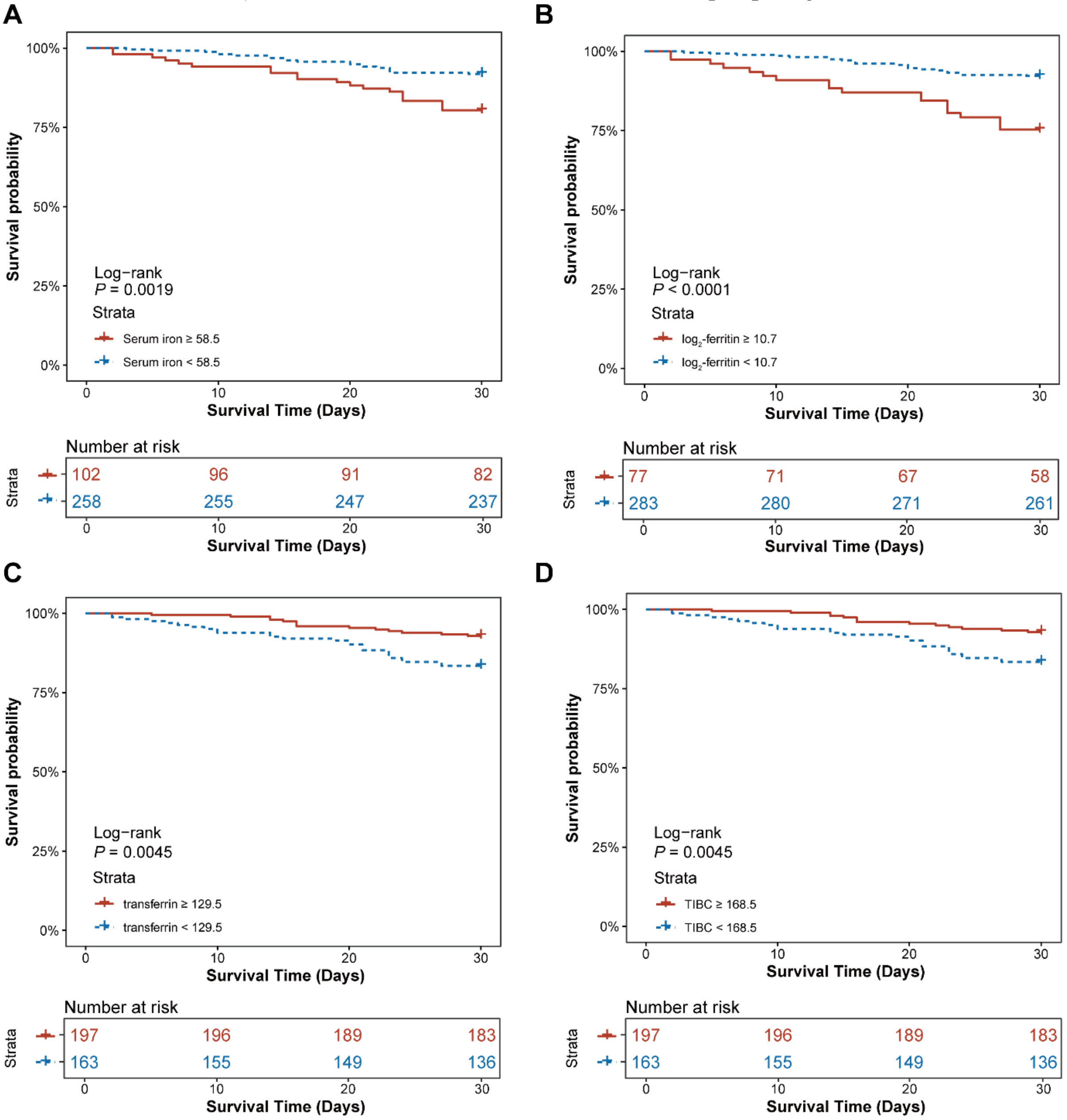

Figure S4. Kaplan-Meier survival analysis curves showing the relationship between iron homeostasis indicators and 90-day all-cause mortality (**A**, Serum iron; **B**, $\log_2$-Ferritin; **C**, Transferrin; **D**, TIBC). Abbreviations: TIBC, total iron binding capacity.

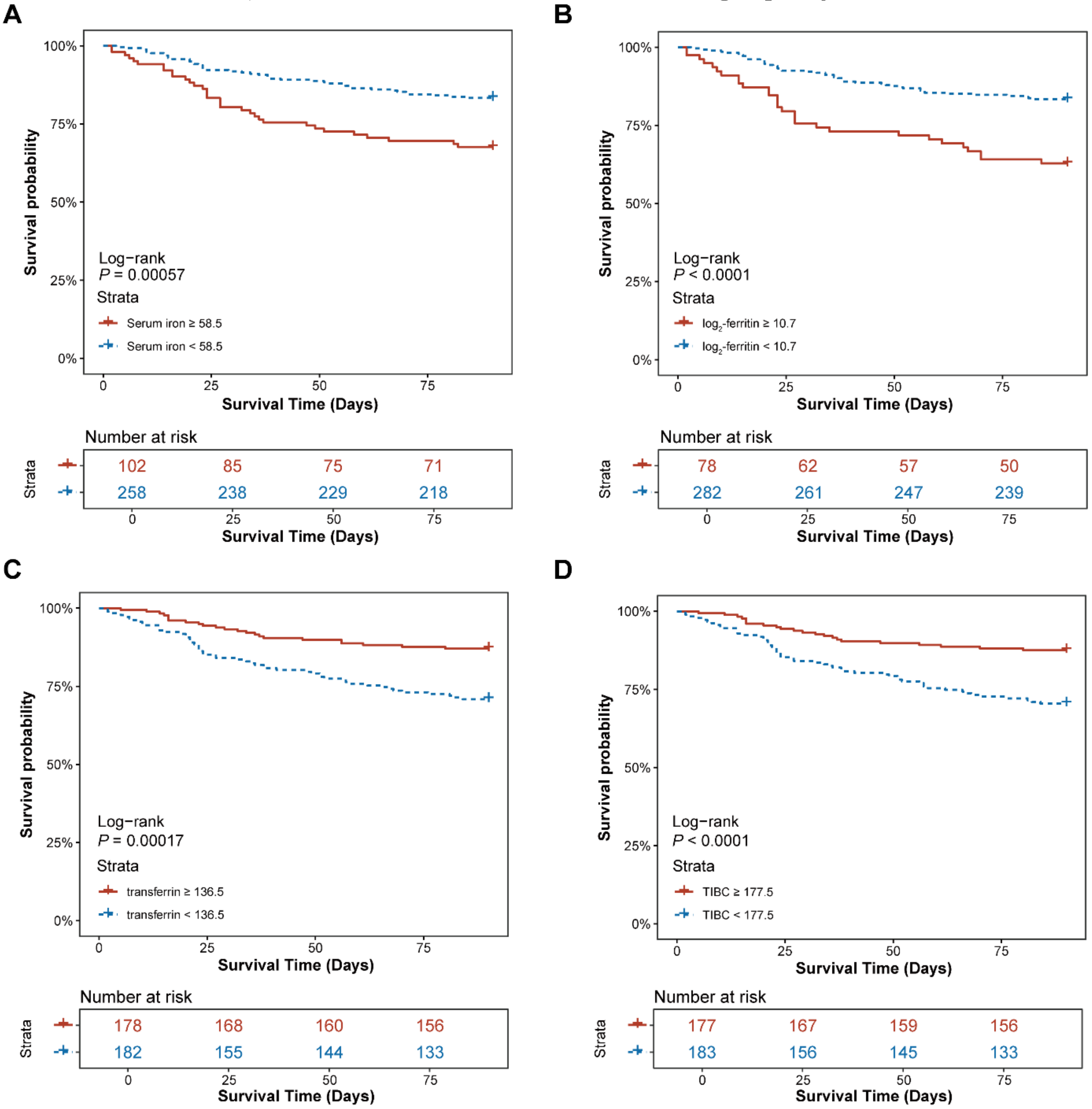

Figure S5. Restricted cubic spline analysis of iron homeostasis indicators and in-hospital all-cause mortality (**A**, Serum iron; **B**, log_2-Ferritin). The blue lines represent the estimated adjusted hazard ratios, while the blue band indicates the corresponding 95% confidence interval. Adjustments follow Model 3. Abbreviations: TIBC, total iron binding capacity.

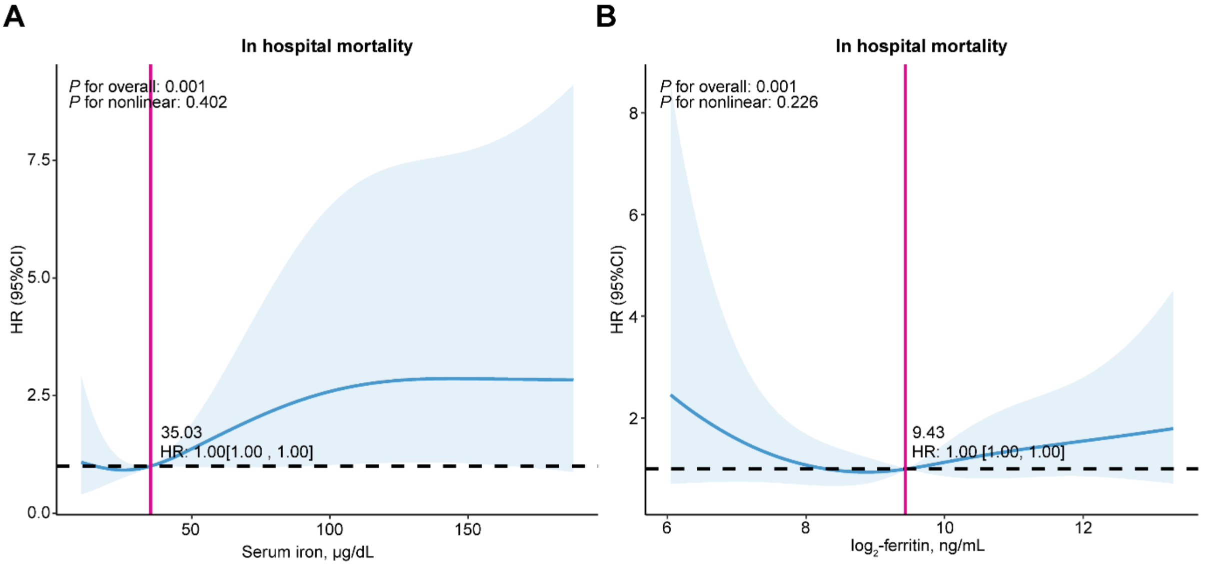

Figure S6. Restricted cubic spline analysis of iron homeostasis indicators and 30-day all-cause mortality (**A**, Serum iron; **B**, log_2-Ferritin; **C**, Transferrin; **D**, TIBC). The blue lines represent the estimated adjusted hazard ratios, while the blue band indicates the corresponding 95% confidence interval. Adjustments follow Model 3. Abbreviations: TIBC, total iron binding capacity.

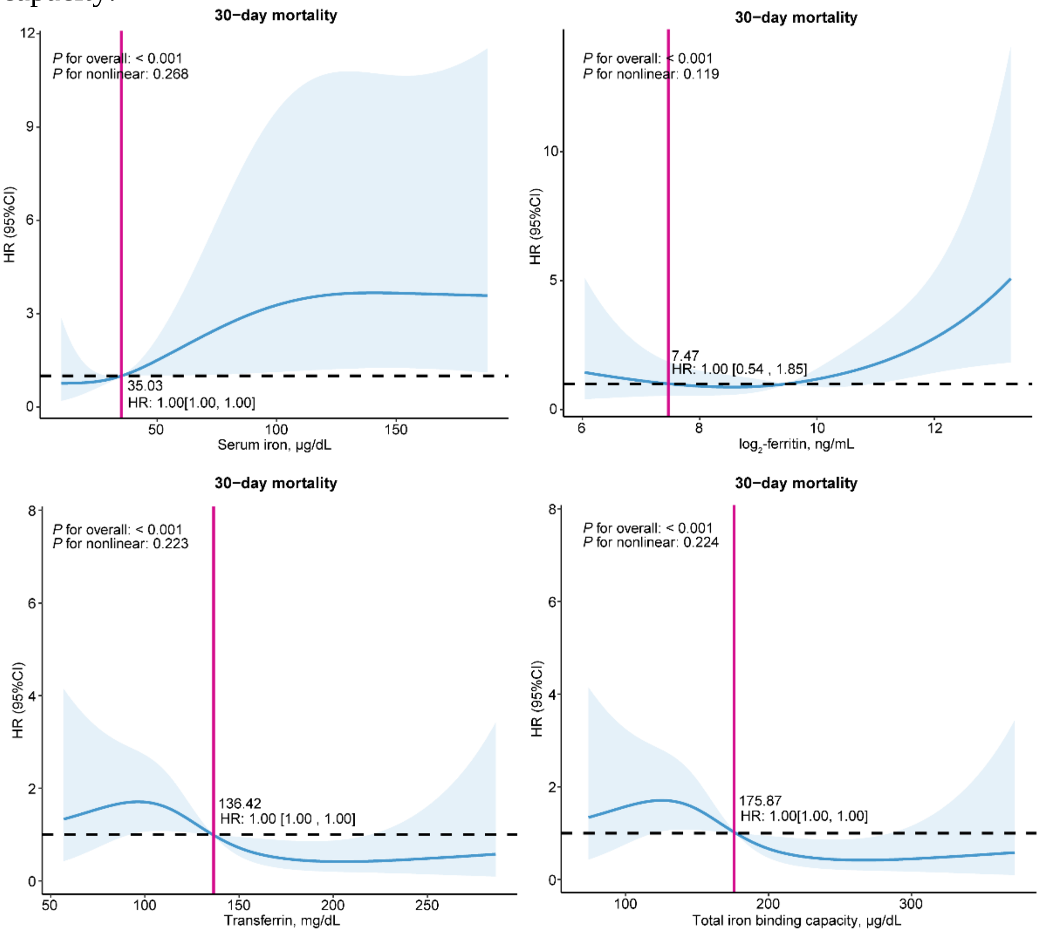

Figure S7. Restricted cubic spline analysis of iron homeostasis indicators and 90-day all-cause mortality (**A**, Serum iron; **B**, log_2-Ferritin). The blue lines represent the estimated adjusted hazard ratios, while the blue band indicates the corresponding 95% confidence interval. Adjustments follow Model 3. Abbreviations: TIBC, total iron binding capacity.

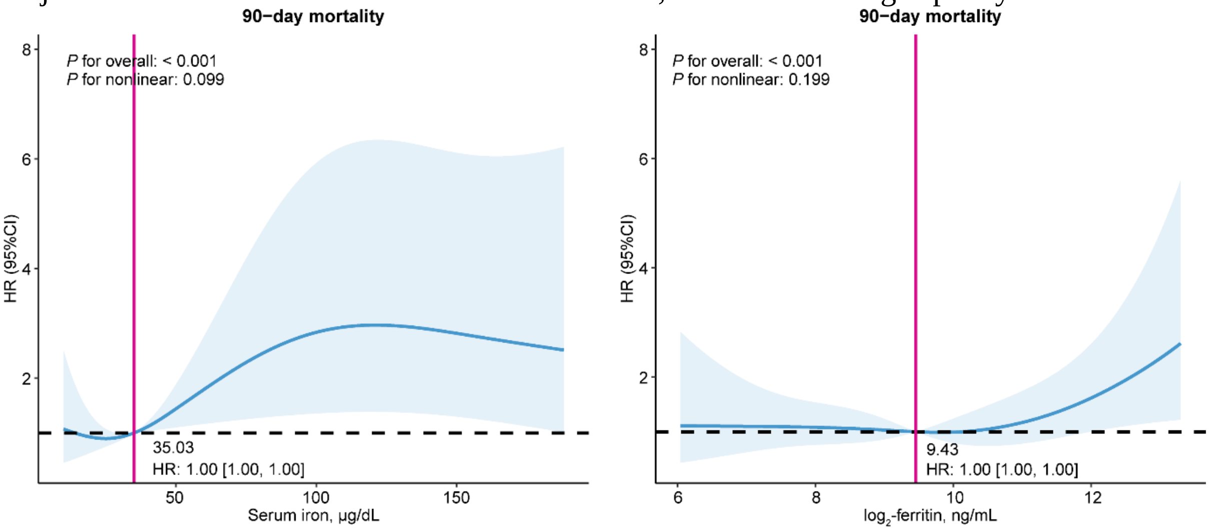

Figure S8. Restricted cubic spline analysis of iron homeostasis indicators and 365-day all-cause mortality (**A**, Serum iron; **B**, log_2-Ferritin; **C**, Transferrin; **D**, TIBC). The blue lines represent the estimated adjusted hazard ratios, while the blue band indicates the corresponding 95% confidence interval. Adjustments follow Model 3.

Abbreviations: TIBC, total iron binding capacity.

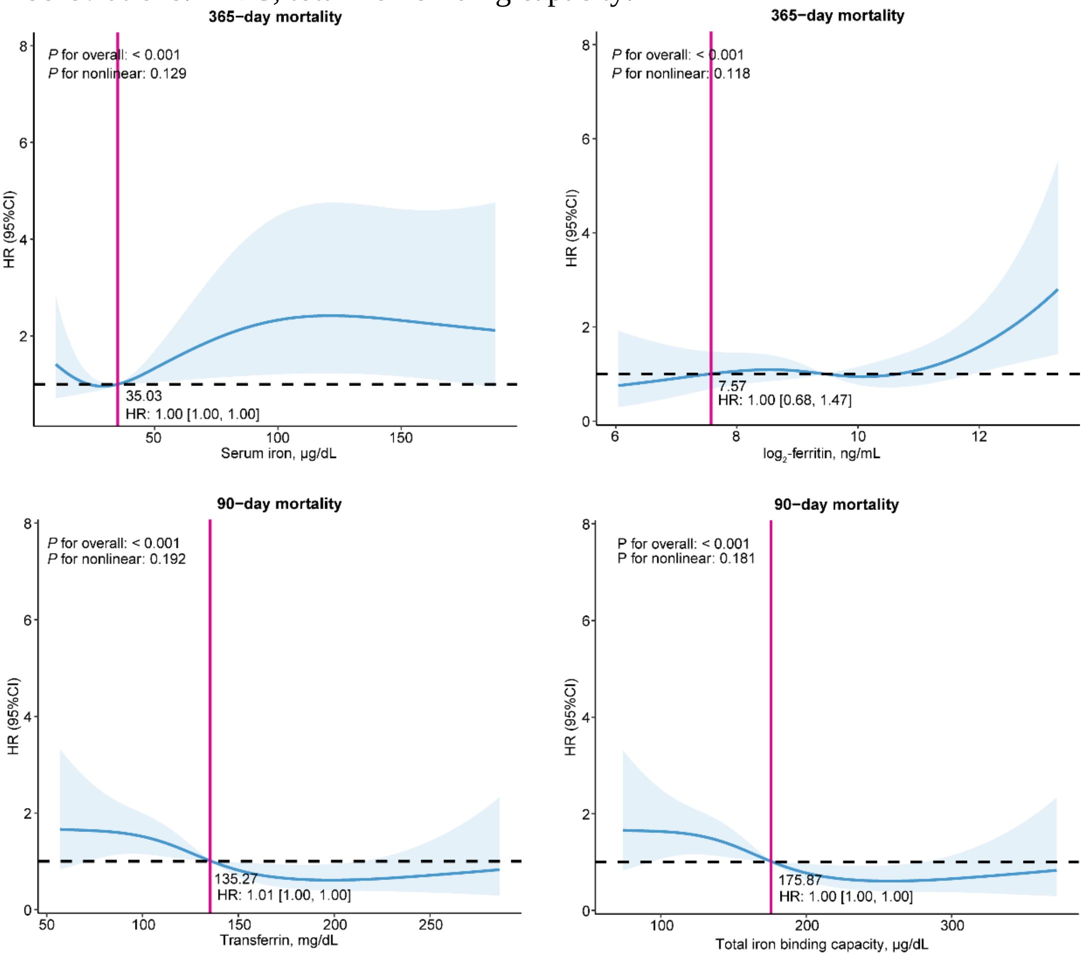

Figure S9. Subgroup and interaction analyses between iron homeostasis indicators and In-hospital mortality (**A**, Serum iron; **B**, log_2-Ferritin; **C**, Transferrin; **D**, TIBC). The adjustment approach is the same as Model 3, except for the stratified variables.
Abbreviations: TIBC, total iron binding capacity.

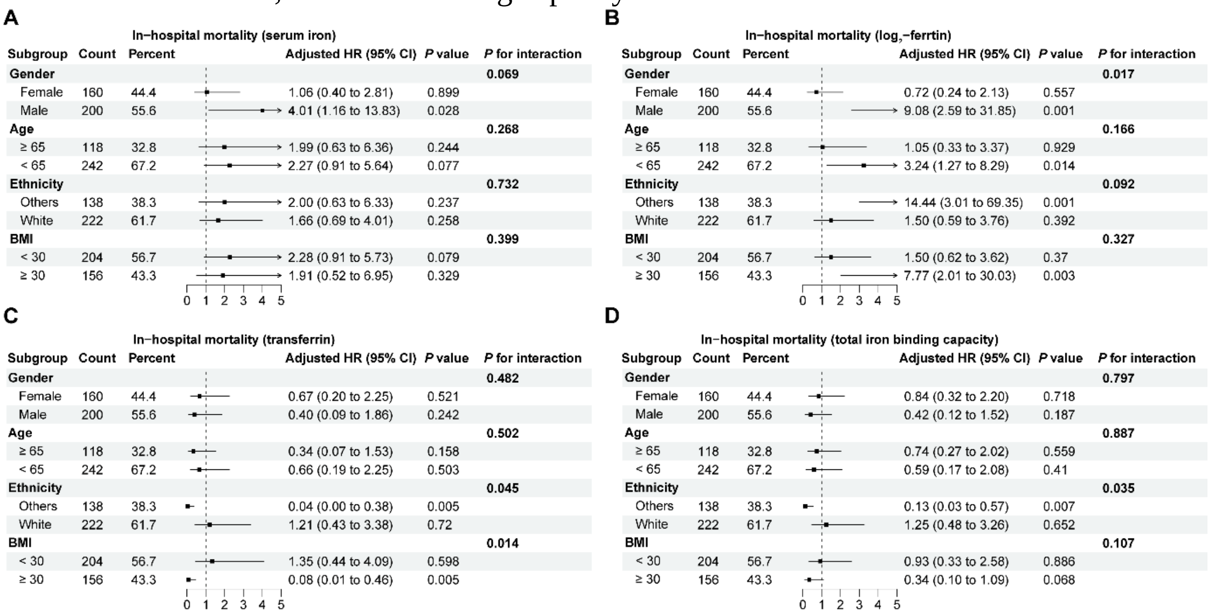

Figure S10. Subgroup and interaction analyses between iron homeostasis indicators and 30-day mortality (**A**, Serum iron; **B**, log_2-Ferritin; **C**, Transferrin; **D**, TIBC). The adjustment approach is the same as Model 3, except for the stratified variables. Abbreviations: TIBC, total iron binding capacity.

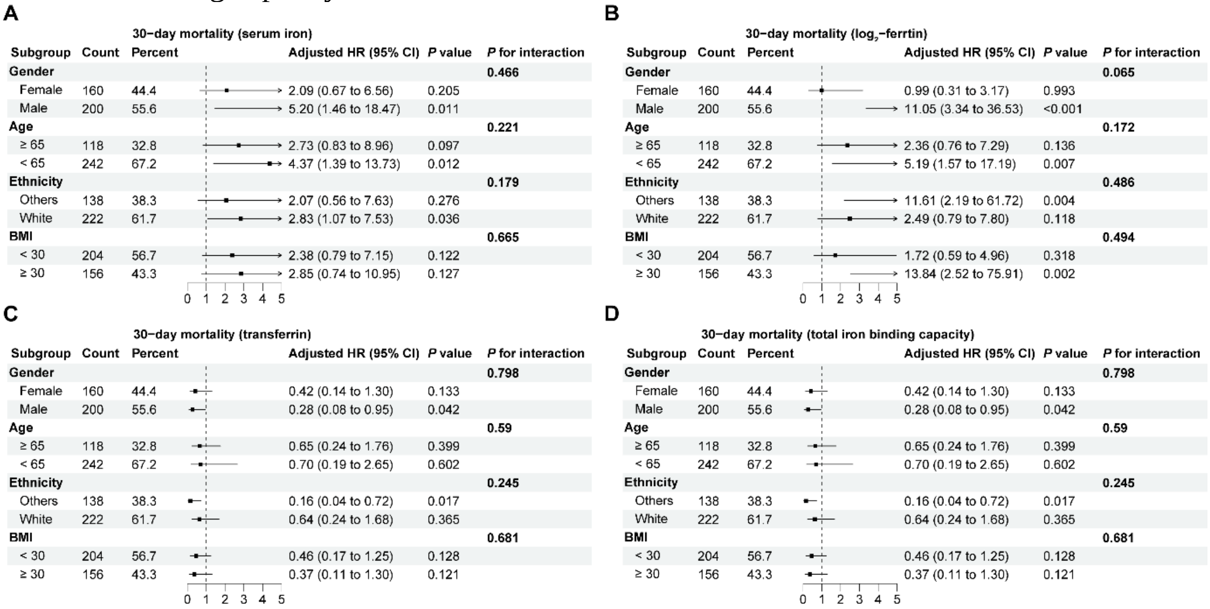

Figure S11. Subgroup and interaction analyses between iron homeostasis indicators and 90-day mortality (**A**, Serum iron; **B**, log_2-Ferritin; **C**, Transferrin; **D**, TIBC). The adjustment approach is the same as Model 3, except for the stratified variables. Abbreviations: TIBC, total iron binding capacity.

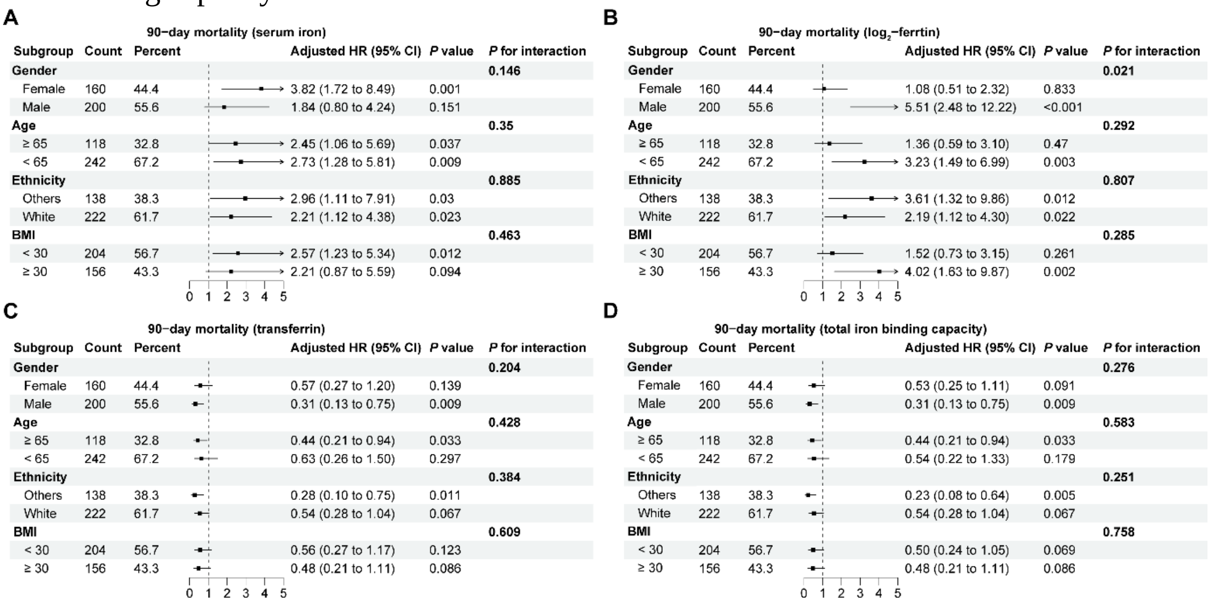

Figure S12. Subgroup and interaction analyses between iron homeostasis indicators and 365-day mortality (**A**, Serum iron; **B**, log_2-Ferritin; **C**, Transferrin; **D**, TIBC). The adjustment approach is the same as Model 3, except for the stratified variables. Abbreviations: TIBC, total iron binding capacity.

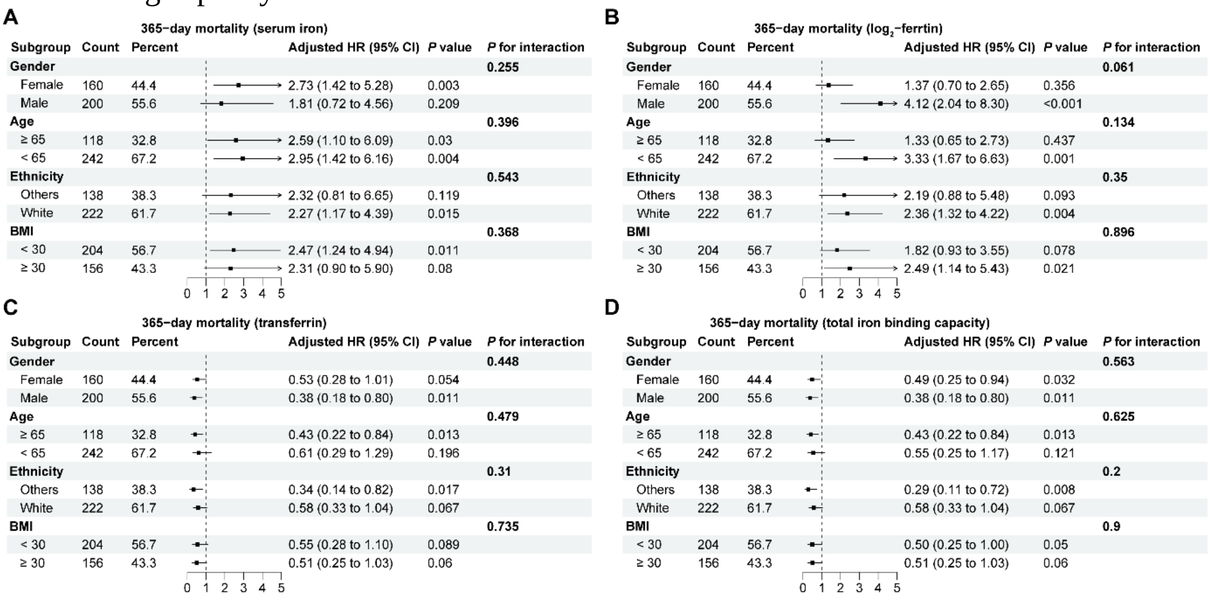

Table S1 Cox proportional hazard models for serum iron

Parameter	Model 1		Model 2		Model 3	
	HR (95%CI)	*P* value	HR (95%CI)	*P* value	HR (95%CI)	*P* value
In-hospital mortality						
Continuous variable per unit, µg/dL	1.006 (1.001 - 1.01)	0.013	1.007 (1.002 - 1.012)	0.005	1.006 (1.000 - 1.012)	0.04
Quartile						
Q1	Ref.		Ref.		Ref.	
Q2	1.531 (0.653 - 3.592)	0.327	1.557 (0.659 - 3.678)	0.312	1.066 (0.424 - 2.681)	0.891
Q3	1.852 (0.772 - 4.442)	0.167	1.852 (0.741 - 4.627)	0.187	1.396 (0.541 - 3.601)	0.491
Q4	2.689 (1.259 - 5.742)	0.011	2.960 (1.353 - 6.475)	0.007	2.340 (0.988 - 5.543)	0.053
30-day mortality						
Continuous variable per unit, µg/dL	1.007 (1.002 - 1.012)	0.005	1.010 (1.004 - 1.015)	<0.001	1.008 (1.002 - 1.014)	0.012
Quartile						
Q1	Ref.		Ref.		Ref.	
Q2	2.562 (0.804 - 8.169)	0.112	2.235 (0.694 - 7.197)	0.178	1.593 (0.462 - 5.498)	0.461
Q3	2.633 (0.826 - 8.395)	0.102	2.526 (0.784 - 8.144)	0.121	1.906 (0.554 - 6.554)	0.306
Q4	4.726 (1.590 - 14.047)	0.005	5.553 (1.851 - 16.661)	0.002	4.098 (1.247 - 13.464)	0.020
90-day mortality						
Continuous variable per unit, µg/dL	1.005 (1.001 - 1.009)	0.012	1.007 (1.003 - 1.011)	0.001	1.006 (1.001 - 1.010)	0.021
Quartile						
Q1	Ref.		Ref.		Ref.	
Q2	1.739 (0.850 - 3.557)	0.130	1.594 (0.774 - 3.283)	0.206	1.333 (0.623 - 2.852)	0.458
Q3	1.430 (0.677 - 3.023)	0.349	1.384 (0.649 - 2.950)	0.401	1.130 (0.506 - 2.522)	0.766
Q4	2.764 (1.405 - 5.436)	0.003	3.207 (1.617 - 6.357)	0.001	2.603 (1.222 - 5.542)	0.013

Model 1: Unadjusted model; Model 2: adjusted for gender, age, race; Model 3: adjusted for gender, age, race, BMI, WBC, Platelet, Glucose, ALT, Creatinine, Anion gap, Heart failure, Hypertension, Diabetes, SOFA, CCI, SAPS II, Chronic pulmonary disease, Malignant cancer, Spesis.

Table S2. Cox proportional hazard models for log_2-ferritin

Parameter	Model 1		Model 2		Model 3	
	HR (95%CI)	*P* value	HR (95%CI)	*P* value	HR (95%CI)	*P* value
In-hospital mortality						
Continuous variable per unit, ng/mL	1.1 (0.943 - 1.284)	0.226	1.092 (0.940 - 1.269)	0.249	1.046 (0.877 - 1.248)	0.617
Quartile						
Q1	Ref.		Ref.		Ref.	
Q2	1.231 (0.546 - 2.774)	0.616	1.039 (0.452 - 2.385)	0.929	0.846 (0.344 - 2.079)	0.716
Q3	0.562 (0.214 - 1.480)	0.244	0.541(0.203 - 1.442)	0.219	0.578 (0.201 - 1.662)	0.309
Q4	1.552 (0.734 - 3.280)	0.250	1.583(0.744 - 3.368)	0.233	1.290 (0.567 - 2.934)	0.544
30-day mortality						
Continuous variable per unit, ng/mL	1.258 (1.059 - 1.494)	0.009	1.314(1.102 - 1.566)	0.002	1.281 (1.051 - 1.561)	0.014
Quartile						
Q1	Ref.		Ref.		Ref.	
Q2	0.900 (0.347 - 2.334)	0.829	0.907 (0.349 - 2.356)	0.841	0.711 (0.248 - 2.043)	0.527
Q3	0.55 (0.184 - 1.640)	0.283	0.533 (0.177 - 1.603)	0.263	0.589 (0.183 - 1.898)	0.375
Q4	2.272 (1.028 - 5.022)	0.043	2.705 (1.213 - 6.031)	0.015	2.300 (0.946 - 5.591)	0.066
90-day mortality						
Continuous variable per unit, ng/mL	1.16 (1.018 - 1.322)	0.026	1.19 (1.046 - 1.354)	0.008	1.117 (0.970 - 1.287)	0.125
Quartile						
Q1	Ref.		Ref.		Ref.	
Q2	0.905 (0.471 - 1.741)	0.765	0.947 (0.492 - 1.826)	0.871	0.838 (0.418 - 1.680)	0.619
Q3	0.559 (0.266 - 1.175)	0.125	0.590 (0.279 - 1.247)	0.167	0.559 (0.257 - 1.216)	0.142
Q4	1.689 (0.947 - 3.013)	0.076	1.962 (1.095 - 3.514)	0.023	1.474 (0.785 - 2.769)	0.227

Model 1: Unadjusted model; Model 2: adjusted for gender, age, race; Model 3: adjusted for gender, age, race, BMI, WBC, Platelet, Glucose, ALT, Creatinine, Anion gap, Heart failure, Hypertension, Diabetes, SOFA, CCI, SAPS II, Chronic pulmonary disease, Malignant cancer, Spesis.

Table S3. Cox proportional hazard models for transferrin

Parameter	Model 1		Model 2		Model 3	
	HR (95%CI)	*P* value	HR (95%CI)	*P* value	HR (95%CI)	*P* value
In-hospital mortality						
Continuous variable per unit, mg/dL	0.999 (0.994 - 1.005)	0.815	1.000 (0.994 - 1.006)	0.965	1.000 (0.994 - 1.006)	0.939
Quartile						
Q1	Ref.		Ref.		Ref.	
Q2	1.036 (0.545 - 1.968)	0.914	0.976 (0.504 - 1.892)	0.943	1.018 (0.486 - 2.129)	0.963
Q3	0.695 (0.320 - 1.511)	0.359	0.672 (0.308 - 1.466)	0.317	0.732 (0.305 - 1.759)	0.485
Q4	0.598 (0.240 - 1.487)	0.269	0.629 (0.252 - 1.572)	0.321	0.470 (0.172 - 1.285)	0.141
30-day mortality						
Continuous variable per unit, mg/dL	0.992 (0.985 - 0.998)	0.013	0.992 (0.985 - 0.999)	0.017	0.992 (0.985 - 1.000)	**0.041**
Quartile						
Q1	Ref.		Ref.		Ref.	
Q2	0.916 (0.441 - 1.905)	0.815	0.898 (0.429 - 1.879)	0.774	0.845 (0.361 - 1.976)	0.697
Q3	0.522 (0.223 - 1.220)	0.133	0.465 (0.197 - 1.093)	0.079	0.575 (0.212 - 1.563)	0.278
Q4	0.251 (0.084 - 0.751)	0.013	0.259 (0.086 - 0.775)	0.016	0.186 (0.053 - 0.653)	**0.009**
90-day mortality						
Continuous variable per unit, mg/dL	0.993 (0.988 - 0.998)	0.003	0.993 (0.988 - 0.998)	0.004	0.995 (0.99 - 1.000)	**0.039**
Quartile						
Q1	Ref.		Ref.		Ref.	
Q2	0.902 (0.525 - 1.55)	0.709	0.905 (0.525 - 1.56)	0.72	1.041 (0.581 - 1.866)	0.892
Q3	0.484 (0.256 - 0.915)	0.026	0.454 (0.239 - 0.862)	0.016	0.660 (0.328 - 1.328)	0.244
Q4	0.291 (0.138 - 0.616)	0.001	0.288 (0.136 - 0.609)	0.001	0.304 (0.138 - 0.672)	**0.003**

Model 1: Unadjusted model; Model 2: adjusted for gender, age, race; Model 3: adjusted for gender, age, race, BMI, WBC, Platelet, Glucose, ALT, Creatinine, Anion gap, Heart failure, Hypertension, Diabetes, SOFA, CCI, SAPS II, Chronic pulmonary disease, Malignant cancer, Spesis

Table S4. Cox proportional hazard models for total iron binding capacity

Parameter	Model 1		Model 2		Model 3	
	HR (95%CI)	*P* value	HR (95%CI)	*P* value	HR (95%CI)	*P* value
In hospital mortality						
Continuous variable per unit, μg/dL	0.999 (0.995 - 1.004)	0.801	1.000 (0.996 - 1.004)	0.978	1.000 (0.995 - 1.004)	0.931
Quartile						
Q1	Ref.		Ref.		Ref.	
Q2	1.076 (0.573 - 2.021)	0.819	1.011 (0.529 - 1.932)	0.974	1.038 (0.502 - 2.143)	0.921
Q3	0.635 (0.282 - 1.427)	0.271	0.618 (0.274 - 1.394)	0.246	0.688 (0.278 - 1.705)	0.419
Q4	0.597 (0.24 - 1.483)	0.266	0.627 (0.251 - 1.567)	0.318	0.472 (0.173 - 1.29)	0.143
30-day mortality						
Continuous variable per unit, μg/dL	0.994 (0.988 - 0.999)	0.013	0.994 (0.989 - 0.999)	0.018	0.994 (0.988 - 1.000)	**0.041**
Quartile						
Q1	Ref.		Ref.		Ref.	
Q2	0.905 (0.435 - 1.881)	0.788	0.878 (0.42 - 1.836)	0.730	0.822 (0.353 - 1.916)	0.650
Q3	0.528 (0.226 - 1.235)	0.141	0.473 (0.201 - 1.113)	0.086	0.595 (0.219 - 1.62)	0.310
Q4	0.251 (0.084 - 0.751)	0.013	0.259 (0.086 - 0.775)	0.016	0.187 (0.053 - 0.655)	**0.009**
90-day mortality						
Continuous variable per unit, μg/dL	0.994 (0.991 - 0.998)	0.003	0.994 (0.991 - 0.998)	0.004	0.996 (0.992 - 1.000)	**0.038**
Quartile						
Q1	Ref.		Ref.		Ref.	
Q2	0.930 (0.545 - 1.588)	0.790	0.931 (0.544 - 1.594)	0.794	1.066 (0.598 - 1.898)	0.829
Q3	0.453 (0.236 - 0.872)	0.018	0.426 (0.22 - 0.822)	0.011	0.621 (0.304 - 1.269)	0.191
Q4	0.291 (0.138 - 0.616)	0.001	0.288 (0.136 - 0.609)	0.001	0.304 (0.138 - 0.672)	**0.003**

Model 1: Unadjusted model; Model 2: adjusted for gender, age, race; Model 3: adjusted for gender, age, race, BMI, WBC, Platelet, Glucose, ALT, Creatinine, Anion gap, Heart failure, Hypertension, Diabetes, SOFA, CCI, SAPS II, Chronic pulmonary disease, Malignant cancer, Spesis

Table S5. Cox proportional hazard models for serum iron using different imputation methods

Parameter	Model 3 (PPM)		Model 3 (Random forest)		Model 3 (norm)	
	HR (95%CI)	*P* value	HR (95%CI)	*P* value	HR (95%CI)	*P* value
In-hospital mortality						
Continuous variable per unit, µg/dL	1.006 (1.000 - 1.012)	0.04	1.006 (1 - 1.012)	0.044	1.006 (1 - 1.012)	0.042
Quartile						
Q1	Ref.		Ref.		Ref.	
Q2	1.066 (0.424 - 2.681)	0.891	1.040 (0.413 - 2.617)	0.933	1.016 (0.403 - 2.559)	0.974
Q3	1.396 (0.541 - 3.601)	0.491	1.394 (0.539 - 3.607)	0.493	1.423 (0.547 - 3.698)	0.469
Q4	2.340 (0.988 - 5.543)	0.053	2.341 (0.993 - 5.521)	0.052	2.305 (0.975 - 5.450)	0.057
30-day mortality						
Continuous variable per unit, µg/dL	1.008 (1.002 - 1.014)	0.012	1.008 (1.002 - 1.014)	0.013	1.008 (1.001 - 1.014)	0.016
Quartile						
Q1	Ref.		Ref.		Ref.	
Q2	1.593 (0.462 - 5.498)	0.461	1.542 (0.444 - 5.357)	0.495	1.565 (0.451 - 5.436)	0.481
Q3	1.906 (0.554 - 6.554)	0.306	1.928 (0.561 - 6.626)	0.297	1.992 (0.576 - 6.89)	0.276
Q4	4.098 (1.247 - 13.464)	0.02	4.021 (1.223 - 13.222)	0.022	3.980 (1.207 - 13.128)	0.023
90-day mortality						
Continuous variable per unit, µg/dL	1.006 (1.001 - 1.010)	0.021	1.005 (1.001 - 1.01)	0.03	1.005 (1.001 - 1.01)	0.029
Quartile						
Q1	Ref.		Ref.		Ref.	
Q2	1.333 (0.623 - 2.852)	0.458	1.293 (0.603 - 2.772)	0.509	1.291 (0.602 - 2.772)	0.512
Q3	1.130 (0.506 - 2.522)	0.766	1.132 (0.509 - 2.521)	0.761	1.169 (0.523 - 2.612)	0.703

Q4	2.603 (1.222 - 5.542)	0.013	2.547 (1.195 - 5.431)	0.015	2.527 (1.183 - 5.399)	0.017
365-day mortality						
Continuous variable per unit, μg/dL	1.004 (0.999 - 1.008)	0.101	1.004 (0.999 - 1.008)	0.104	1.003 (0.999 - 1.008)	0.125
Quartile						
Q1	Ref.				Ref.	
Q2	1.075 (0.576 - 2.008)	0.82	1.039 (0.555 - 1.947)	0.905	1.058 (0.565 - 1.98)	0.861
Q3	0.815 (0.418 - 1.589)	0.547	0.826 (0.425 - 1.606)	0.573	0.859 (0.44 - 1.675)	0.655
Q4	1.725 (0.915 - 3.253)	0.092	1.705 (0.904 - 3.215)	0.099	1.657 (0.877 - 3.133)	0.12

Model 3: adjusted for gender, age, race, BMI, WBC, Platelet, Glucose, ALT, Creatinine, Anion gap, Heart failure, Hypertension, Diabetes, SOFA, CCI, SAPS II, Chronic pulmonary disease, Malignant cancer, Spesis. Abbreviations: TIBC, total iron binding capacity; CI, confidence interval; HR, hazard ratio; PPM, predictive mean matching.

Table S6. Cox proportional hazard models for log_2-ferritin using different imputation methods

Parameter	Model 3 (PPM)		Model 3 (Random forest)		Model 3 (norm)	
	HR (95%CI)	*P* value	HR (95%CI)	*P* value	HR (95%CI)	*P* value
In-hospital mortality						
Continuous variable per unit, ng/mL	1.046 (0.877 - 1.248)	0.617	1.055 (0.884 - 1.259)	0.556	1.052 (0.881 - 1.255)	0.578
Quartile						
Q1	Ref.		Ref.		Ref.	
Q2	0.846 (0.344 - 2.079)	0.716	0.876 (0.356 - 2.159)	0.774	0.853 (0.346 - 2.1)	0.729
Q3	0.578 (0.201 - 1.662)	0.309	0.599 (0.207 - 1.738)	0.346	0.588 (0.202 - 1.707)	0.328
Q4	1.290 (0.567 - 2.934)	0.544	1.381 (0.601 - 3.176)	0.447	1.343 (0.586 - 3.082)	0.486
30-day mortality						
Continuous variable per unit, ng/mL	1.281 (1.051 - 1.561)	0.014	1.291 (1.056 - 1.577)	0.013	1.282 (1.051 - 1.564)	0.014

Quartile						
Q1	Ref.		Ref.		Ref.	
Q2	0.711 (0.248 - 2.043)	0.527	0.745 (0.258 - 2.151)	0.586	0.727 (0.253 - 2.087)	0.553
Q3	0.589 (0.183 - 1.898)	0.375	0.604 (0.186 - 1.959)	0.401	0.590 (0.182 - 1.910)	0.379
Q4	2.300 (0.946 - 5.591)	0.066	2.438 (0.990 - 6.003)	0.052	2.350 (0.964 - 5.732)	0.060
90-day mortality						
Continuous variable per unit, ng/mL	1.117 (0.970 - 1.287)	0.125	1.115 (0.968 - 1.285)	0.130	1.121 (0.973 - 1.291)	0.113
Quartile						
Q1	Ref.		Ref.		Ref.	
Q2	0.838 (0.418 - 1.680)	0.619	0.855 (0.427 - 1.713)	0.659	0.835 (0.417 - 1.673)	0.611
Q3	0.559 (0.257 - 1.216)	0.142	0.574 (0.264 - 1.248)	0.161	0.574 (0.263 - 1.252)	0.163
Q4	1.474 (0.785 - 2.769)	0.227	1.520 (0.805 - 2.871)	0.197	1.505 (0.800 - 2.833)	0.205
365-day mortality						
Continuous variable per unit, ng/mL	1.157 (1.021 - 1.311)	0.022	1.164 (1.027 - 1.319)	0.017	1.162 (1.026 - 1.316)	0.018
Quartile						
Q1	Ref.		Ref.		Ref.	
Q2	1.156 (0.627 - 2.13)	0.643	1.183 (0.642 - 2.181)	0.59	1.152 (0.625 - 2.122)	0.651
Q3	0.802 (0.416 - 1.548)	0.511	0.830 (0.429 - 1.605)	0.58	0.827 (0.427 - 1.601)	0.573
Q4	1.701 (0.956 - 3.025)	0.071	1.797 (1.006 - 3.209)	0.048	1.741 (0.978 - 3.098)	0.059

Model 3: adjusted for gender, age, race, BMI, WBC, Platelet, Glucose, ALT, Creatinine, Anion gap, Heart failure, Hypertension, Diabetes, SOFA, CCI, SAPS II, Chronic pulmonary disease, Malignant cancer, Spesis. Abbreviations: TIBC, total iron binding capacity; CI, confidence interval; HR, hazard ratio; PPM, predictive mean matching.

Table S7. Cox proportional hazard models for transferrin using different imputation methods

Parameter	Model 3 (PPM)		Model 3 (Random forest)		Model 3 (norm)	
	HR (95%CI)	*P* value	HR (95%CI)	*P* value	HR (95%CI)	*P* value
**In-hospital mortality**						
Continuous variable per unit, mg/dL	1.000 (0.994 - 1.006)	0.939	1.000 (0.994 - 1.006)	0.990	1.000 (0.994 - 1.006)	0.934
Quartile						
Q1	Ref.		Ref.		Ref.	
Q2	1.018 (0.486 - 2.129)	0.963	1.025 (0.497 - 2.115)	0.947	1.016 (0.489 - 2.113)	0.965
Q3	0.732 (0.305 - 1.759)	0.485	0.733 (0.306 - 1.757)	0.487	0.718 (0.298 - 1.732)	0.461
Q4	0.470 (0.172 - 1.285)	0.141	0.494 (0.182 - 1.341)	0.166	0.467 (0.171 - 1.276)	0.138
**30-day mortality**						
Continuous variable per unit, mg/dL	0.992 (0.985 - 1.000)	0.041	0.992 (0.984 - 0.999)	0.037	0.992 (0.984 - 1.000)	0.041
Quartile						
Q1	Ref.		Ref.		Ref.	
Q2	0.845 (0.361 - 1.976)	0.697	0.860 (0.371 - 1.995)	0.726	0.848 (0.364 - 1.978)	0.703
Q3	0.575 (0.212 - 1.563)	0.278	0.527 (0.192 - 1.451)	0.215	0.556 (0.203 - 1.521)	0.253
Q4	0.186 (0.053 - 0.653)	0.009	0.185 (0.053 - 0.645)	0.008	0.189 (0.054 - 0.660)	0.009
**90-day mortality**						
Continuous variable per unit, mg/dL	0.995 (0.99 - 1.000)	0.039	0.995 (0.99 - 1)	0.036	0.995 (0.99 - 1.000)	0.035
Quartile						
Q1	Ref.		Ref.		Ref.	
Q2	1.041 (0.581 - 1.866)	0.892	1.017 (0.569 - 1.818)	0.953	1.034 (0.578 - 1.85)	0.909
Q3	0.660 (0.328 - 1.328)	0.244	0.619 (0.305 - 1.252)	0.182	0.636 (0.316 - 1.277)	0.203
Q4	0.304 (0.138 - 0.672)	0.003	0.304 (0.138 - 0.671)	0.003	0.302 (0.137 - 0.666)	0.003

**365-day mortality**						
Continuous variable per unit, mg/dL	0.994(0.989 - 0.998)	0.007	0.994 (0.989 - 0.998)	0.008	0.994 (0.989 - 0.998)	0.007
Quartile						
Q1	Ref.		Ref.		Ref.	
Q2	0.837 (0.491 - 1.426)	0.513	0.830 (0.488 - 1.41)	0.490	0.834 (0.491 - 1.419)	0.504
Q3	0.666 (0.370 - 1.199)	0.175	0.644 (0.356 - 1.164)	0.145	0.647 (0.361 - 1.163)	0.146
Q4	0.343 (0.179 - 0.658)	0.001	0.351 (0.183 - 0.674)	0.002	0.345 (0.18 - 0.661)	0.001

Model 3: adjusted for gender, age, race, BMI, WBC, Platelet, Glucose, ALT, Creatinine, Anion gap, Heart failure, Hypertension, Diabetes, SOFA, CCI, SAPS II, Chronic pulmonary disease, Malignant cancer, Spesis. Abbreviations: TIBC, total iron binding capacity; CI, confidence interval; HR, hazard ratio; PPM, predictive mean matching.

Table S8. Cox proportional hazard models for total iron binding capacity using different imputation methods

Parameter	Model 3 (PPM)		Model 3 (Random forest)		Model 3 (norm)	
	HR (95%CI)	*P* value	HR (95%CI)	*P* value	HR (95%CI)	*P* value
**In-hospital mortality**						
Continuous variable per unit, μg/dL	1.000 (0.995 - 1.004)	0.931	1.000 (0.995 - 1.005)	0.980	1.000 (0.995 - 1.004)	0.926
Quartile						
Q1	Ref.		Ref.			
Q2	1.038 (0.502 - 2.143)	0.921	1.055 (0.515 - 2.164)	0.884	1.034 (0.503 - 2.124)	0.927
Q3	0.688 (0.278 - 1.705)	0.419	0.679 (0.272 - 1.692)	0.406	0.676 (0.272 - 1.68)	0.4
Q4	0.472 (0.173 - 1.29)	0.143	0.496 (0.183 - 1.348)	0.169	0.468 (0.171 - 1.281)	0.140
**30-day mortality**						
Continuous variable per unit, μg/dL	0.994 (0.988 - 1)	0.041	0.994 (0.988 - 1)	0.04	0.994 (0.988 - 1.000)	0.041

Quartile						
Q1	Ref.		Ref.			
Q2	0.822 (0.353 - 1.916)	0.65	0.819 (0.353 - 1.899)	0.641	0.825 (0.355 - 1.917)	0.654
Q3	0.595 (0.219 - 1.62)	0.31	0.570 (0.206 - 1.577)	0.279	0.576 (0.210 - 1.577)	0.283
Q4	0.187 (0.053 - 0.655)	0.009	0.188 (0.054 - 0.658)	0.009	0.189 (0.054 - 0.663)	0.009
**90-day mortality**						
Continuous variable per unit, μg/dL	0.996 (0.992 - 1.000)	0.038	0.996 (0.992 - 1.000)	0.037	0.996 (0.992 - 1.000)	0.034
Quartile						
Q1	Ref.		Ref.		Ref.	
Q2	1.066 (0.598 - 1.898)	0.829	1.037 (0.584 - 1.842)	0.902	1.058 (0.595 - 1.881)	0.848
Q3	0.621 (0.304 - 1.269)	0.191	0.588 (0.285 - 1.213)	0.15	0.599 (0.294 - 1.221)	0.158
Q4	0.304 (0.138 - 0.672)	0.003	0.305 (0.138 - 0.674)	0.003	0.302 (0.137 - 0.666)	0.003
**365-day mortality**						
Continuous variable per unit, μg/dL	0.995 (0.992 - 0.999)	0.007	0.995 (0.992 - 0.999)	0.008	0.995 (0.992 - 0.999)	0.007
Quartile						
Q1	Ref.		Ref.		Ref.	
Q2	0.858 (0.506 - 1.454)	0.569	0.846 (0.501 - 1.43)	0.532	0.855 (0.506 - 1.445)	0.558
Q3	0.640 (0.352 - 1.161)	0.142	0.625 (0.342 - 1.142)	0.126	0.622 (0.343 - 1.126)	0.117
Q4	0.343 (0.179 - 0.657)	0.001	0.352 (0.183 - 0.674)	0.002	0.345 (0.180 - 0.661)	0.001

Model 3: adjusted for gender, age, race, BMI, WBC, Platelet, Glucose, ALT, Creatinine, Anion gap, Heart failure, Hypertension, Diabetes, SOFA, CCI, SAPS II, Chronic pulmonary disease, Malignant cancer, Spesis. Abbreviations: TIBC, total iron binding capacity; CI, confidence interval; HR, hazard ratio; PPM, predictive mean matching.

Table S9. Cox proportional hazard models for serum iron in Model 3 (Full vs. Stepwise regression)

Parameter	Model 3 (Full model)		Model 3 (Stepwise regression)	
	HR (95%CI)	*P* value	HR (95%CI)	*P* value
**In-hospital mortality**				
Continuous variable per unit, μg/dL	1.006 (1.000 - 1.012)	0.042	1.008 (1.003 - 1.014)	0.004
Quartile				
Q1	Ref.		Ref.	
Q2	1.016 (0.403 - 2.559)	0.974	Not retained by stepwise selection (AIC)	
Q3	1.423 (0.547 - 3.698)	0.469	Not retained by stepwise selection (AIC)	
Q4	2.305 (0.975 - 5.450)	0.057	Not retained by stepwise selection (AIC)	
**30-day mortality**				
Continuous variable per unit, μg/dL	1.008 (1.001 - 1.014)	0.016	1.008 (1.002 - 1.013)	0.007
Quartile				
Q1	Ref.		Ref.	
Q2	1.565 (0.451 - 5.436)	0.481	1.605 (0.496 - 5.197)	0.43
Q3	1.992 (0.576 - 6.890)	0.276	1.941 (0.602 - 6.260)	0.267
Q4	3.980 (1.207 - 13.128)	0.023	4.069 (1.316 - 12.582)	0.015
**90-day mortality**				
Continuous variable per unit, μg/dL	1.005 (1.001 - 1.010)	0.029	1.006 (1.001 - 1.010)	0.009
Quartile				
Q1	Ref.		Ref.	
Q2	1.291 (0.602 - 2.772)	0.512	1.259 (0.611 - 2.595)	0.533
Q3	1.169 (0.523 - 2.612)	0.703	1.129 (0.530 - 2.403)	0.753
Q4	2.527 (1.183 - 5.399)	0.017	2.577 (1.268 - 5.237)	0.009

**365-day mortality**				
Continuous variable per unit, μg/dL	1.003 (0.999 - 1.008)	0.125	1.004 (1.000 - 1.008)	0.071
Quartile				
Q1	Ref.		Ref.	
Q2	1.058 (0.565 - 1.98)	0.861	Not retained by stepwise selection (AIC)	
Q3	0.859 (0.44 - 1.675)	0.655	Not retained by stepwise selection (AIC)	
Q4	1.657 (0.877 - 3.133)	0.120	Not retained by stepwise selection (AIC)	

Model 3: adjusted for gender, age, race, BMI, WBC, Platelet, Glucose, ALT, Creatinine, Anion gap, Heart failure, Hypertension, Diabetes, SOFA, CCI, SAPS II, Chronic pulmonary disease, Malignant cancer, Spesis. Abbreviations: TIBC, total iron binding capacity; CI, confidence interval; HR, hazard ratio; AIC, akaike information criterion.

Table S10. Cox proportional hazard models for log_2-ferritin in Model 3 (Full vs. Stepwise regression)

Parameter	Model 3 (Full model)		Model 3 (Stepwise regression)	
	HR (95%CI)	*P* value	HR (95%CI)	*P* value
**In-hospital mortality**				
Continuous variable per unit, ng/mL	1.046 (0.877 - 1.248)	0.617	Not retained by stepwise selection (AIC)	
Quartile				
Q1	Ref.		Ref.	
Q2	0.846 (0.344 - 2.079)	0.716	Not retained by stepwise selection (AIC)	
Q3	0.578 (0.201 - 1.662)	0.309	Not retained by stepwise selection (AIC)	
Q4	1.290 (0.567 - 2.934)	0.544	Not retained by stepwise selection (AIC)	
**30-day mortality**				
Continuous variable per unit, ng/mL	1.281 (1.051 - 1.561)	0.014	1.246 (1.048 - 1.482)	0.013
Quartile				

Q1	Ref.		Ref.	
Q2	0.711 (0.248 - 2.043)	0.527	0.882 (0.318 - 2.448)	0.810
Q3	0.589 (0.183 - 1.898)	0.375	0.510 (0.164 - 1.587)	0.245
Q4	2.300 (0.946 - 5.591)	0.066	2.141 (0.900 - 5.094)	0.085
**90-day mortality**				
Continuous variable per unit, ng/mL	1.117 (0.970 - 1.287)	0.125	1.122 (0.981 - 1.284)	0.093
Quartile				
Q1	Ref.		Ref.	
Q2	0.838 (0.418 - 1.680)	0.619	0.826 (0.423 - 1.610)	0.574
Q3	0.559 (0.257 - 1.216)	0.142	0.536 (0.253 - 1.133)	0.103
Q4	1.474 (0.785 - 2.769)	0.227	1.453 (0.796 - 2.650)	0.224
**365-day mortality**				
Continuous variable per unit, ng/mL	1.157 (1.021 - 1.311)	0.022	1.153 (1.022 - 1.300)	0.020
Quartile				
Q1	Ref.		Ref.	
Q2	1.156 (0.627 - 2.130)	0.643	1.089 (0.606 - 1.959)	0.775
Q3	0.802 (0.416 - 1.548)	0.511	0.787 (0.414 - 1.496)	0.464
Q4	1.701 (0.956 - 3.025)	0.071	1.644 (0.946 - 2.860)	0.078

Model 3: adjusted for gender, age, race, BMI, WBC, Platelet, Glucose, ALT, Creatinine, Anion gap, Heart failure, Hypertension, Diabetes, SOFA, CCI, SAPS II, Chronic pulmonary disease, Malignant cancer, Spesis. Abbreviations: TIBC, total iron binding capacity; CI, confidence interval; HR, hazard ratio; AIC, akaike information criterion.

Table S11. Cox proportional hazard models for transferrin in Model 3 (Full vs. Stepwise regression)

Parameter	Model 3 (Full model)		Model 3 (stepwise regression)	
	HR (95%CI)	*P* value	HR (95%CI)	*P* value
**In-hospital mortality**				
Continuous variable per unit, mg/dL	1.000 (0.994 - 1.006)	0.939	Not retained by stepwise selection (AIC)	
Quartile				
Q1	Ref.		Ref.	
Q2	1.018 (0.486 - 2.129)	0.963	Not retained by stepwise selection (AIC)	
Q3	0.732 (0.305 - 1.759)	0.485	Not retained by stepwise selection (AIC)	
Q4	0.470 (0.172 - 1.285)	0.141	Not retained by stepwise selection (AIC)	
**30-day mortality**				
Continuous variable per unit, mg/dL	0.992 (0.985 - 1)	0.041	0.993 (0.986 - 1)	0.039
Quartile				
Q1	Ref.		Ref.	
Q2	0.845 (0.361 - 1.976)	0.697	0.906 (0.415 - 1.977)	0.803
Q3	0.575 (0.212 - 1.563)	0.278	0.580 (0.236 - 1.422)	0.234
Q4	0.186 (0.053 - 0.653)	0.009	0.193 (0.059 - 0.632)	0.007
**90-day mortality**				
Continuous variable per unit, mg/dL	0.995 (0.99 - 1)	0.039	0.995 (0.99 - 0.999)	0.029
Quartile				
Q1	Ref.		Ref.	
Q2	1.041 (0.581 - 1.866)	0.892	1.070 (0.612 - 1.871)	0.813
Q3	0.660 (0.328 - 1.328)	0.244	0.654 (0.336 - 1.272)	0.211
Q4	0.304 (0.138 - 0.672)	0.003	0.324 (0.151 - 0.696)	0.004

**365-day mortality**				
Continuous variable per unit, mg/dL	0.994(0.989-0.998)	0.007	0.994 (0.99 - 0.998)	0.004
Quartile				
Q1	Ref.		Ref.	
Q2	0.837 (0.491 - 1.426)	0.513	0.841 (0.503 - 1.407)	0.509
Q3	0.666 (0.370 - 1.199)	0.175	0.658 (0.375 - 1.157)	0.146
Q4	0.343 (0.179 - 0.658)	0.001	0.353 (0.189 - 0.66)	0.001

Model 3: adjusted for gender, age, race, BMI, WBC, Platelet, Glucose, ALT, Creatinine, Anion gap, Heart failure, Hypertension, Diabetes, SOFA, CCI, SAPS II, Chronic pulmonary disease, Malignant cancer, Spesis. Abbreviations: TIBC, total iron binding capacity; CI, confidence interval; HR, hazard ratio; AIC, akaike information criterion.

Table S12. Cox proportional hazard models for total iron binding capacity in Model 3 (Full vs. Stepwise regression)

Parameter	Model 3 (Full model)		Model 3 (stepwise regression)	
	HR (95%CI)	*P* value	HR (95%CI)	*P* value
**In-hospital mortality**				
Continuous variable per unit, μg/dL	1.000 (0.995 - 1.004)	0.931	Not retained by stepwise selection (AIC)	
Quartile				
Q1	Ref.			
Q2	1.038 (0.502 - 2.143)	0.921	Not retained by stepwise selection (AIC)	
Q3	0.688 (0.278 - 1.705)	0.419	Not retained by stepwise selection (AIC)	
Q4	0.472 (0.173 - 1.290)	0.143	Not retained by stepwise selection (AIC)	
**30-day mortality**				
Continuous variable per unit, μg/dL	0.994 (0.988 - 1.000)	0.041	0.995 (0.989 - 1.000)	0.039
Quartile				
Q1	Ref.		Ref.	

Q2	0.822 (0.353 - 1.916)	0.65	0.875 (0.401 - 1.906)	0.736
Q3	0.595 (0.219 - 1.620)	0.31	0.601 (0.245 - 1.476)	0.267
Q4	0.187 (0.053 - 0.655)	0.009	0.194 (0.059 - 0.635)	0.007
**90-day mortality**				
Continuous variable per unit, µg/dL	0.996 (0.992 - 1.000)	0.038	0.996 (0.992 - 1.000)	0.028
Quartile				
Q1	Ref.		Ref.	
Q2	1.066 (0.598 - 1.898)	0.829	1.097 (0.631 - 1.906)	0.743
Q3	0.621 (0.304 - 1.269)	0.191	0.615 (0.311 - 1.216)	0.162
Q4	0.304 (0.138 - 0.672)	0.003	0.323 (0.150 - 0.695)	0.004
**365-day mortality**				
Continuous variable per unit, µg/dL	0.995 (0.992 - 0.999)	0.007	0.995 (0.992 - 0.999)	0.004
Quartile				
Q1	Ref.		Ref.	
Q2	0.858 (0.506 - 1.454)	0.569	0.862 (0.518 - 1.433)	0.566
Q3	0.640 (0.352 - 1.161)	0.142	0.633 (0.357 - 1.122)	0.118
Q4	0.343 (0.179 - 0.657)	0.001	0.352 (0.188 - 0.659)	0.001

Model 3: adjusted for gender, age, race, BMI, WBC, Platelet, Glucose, ALT, Creatinine, Anion gap, Heart failure, Hypertension, Diabetes, SOFA, CCI, SAPS II, Chronic pulmonary disease, Malignant cancer, Spesis. Abbreviations: TIBC, total iron binding capacity; CI, confidence interval; HR, hazard ratio; AIC, akaike information criterion.
